# A Diverged Transcriptional Network for Usage of Two Fe-S Cluster Biogenesis Machineries in the Delta-Proteobacterium Myxococcus xanthus

**DOI:** 10.1128/mbio.03001-22

**Published:** 2023-01-19

**Authors:** Mathieu Sourice, Isabel Askenasy, Pierre Simon Garcia, Yann Denis, Gaël Brasseur, Patricia J. Kiley, Béatrice Py, Corinne Aubert

**Affiliations:** a Laboratoire de Chimie Bactérienne (UMR7283), Institut de Microbiologie de la Méditerranée, Institut Microbiologie Bioénergies et Biotechnologie, Centre National de la Recherche Scientifique, Aix-Marseille Université, Marseille, France; b Department of Biomolecular Chemistry, University of Wisconsin–Madison, Madison, Wisconsin, USA; c Department of Microbiology, Unit Stress Adaptation and Metabolism in Enterobacteria, Institut Pasteur, Université Paris Cité, Paris, France; d Department of Microbiology, Unit Evolutionary Biology of the Microbial Cell, Institut Pasteur, Université Paris Cité, Paris, France; e Plate-forme Transcriptomique, Institut de Microbiologie de la Méditerranée (FR3479), Marseille, France; Michigan State University; California Institute of Technology

**Keywords:** Fe-S cluster biogenesis, Fe-S cluster homeostasis, *Myxococcus xanthus*, Rrf2-type regulator, transcription regulation, oxidative stress, iron starvation

## Abstract

Myxococcus xanthus possesses two Fe-S cluster biogenesis machineries, ISC (iron-sulfur cluster) and SUF (sulfur mobilization). Here, we show that in comparison to the phylogenetically distant Enterobacteria, which also have both machineries, M. xanthus evolved an independent transcriptional scheme to coordinately regulate the expression of these machineries. This transcriptional response is directed by RisR, which we show to belong to a phylogenetically distant and biochemically distinct subgroup of the Rrf2 transcription factor family, in comparison to IscR that regulates the *isc* and *suf* operons in Enterobacteria. We report that RisR harbors an Fe-S cluster and that holo-RisR acts as a repressor of both the *isc* and *suf* operons, in contrast to Escherichia coli, where holo-IscR represses the *isc* operon whereas apo-IscR activates the *suf* operon. In addition, we establish that the nature of the cluster and the DNA binding sites of RisR, in the *isc* and *suf* operons, diverge from those of IscR. We further show that in M. xanthus, the two machineries appear to be fully interchangeable in maintaining housekeeping levels of Fe-S cluster biogenesis and in synthesizing the Fe-S cluster for their common regulator, RisR. We also demonstrate that in response to oxidative stress and iron limitation, transcriptional upregulation of the M. xanthus *isc* and *suf* operons was mediated solely by RisR and that the contribution of the SUF machinery was greater than the ISC machinery. Altogether, these findings shed light on the diversity of homeostatic mechanisms exploited by bacteria to coordinately use two Fe-S cluster biogenesis machineries.

## INTRODUCTION

Iron-sulfur (Fe-S) clusters are ancient cofactors that are found in the three domains of life and in viruses. A wide variety of essential biological processes such as respiration, photosynthesis, amino acid and cofactor biosynthesis, DNA repair, tRNA modification, and gene regulation rely on Fe-S cluster-based chemistry (1 to 4). In addition, many secondary-metabolite biosynthetic pathways involve Fe-S-containing proteins whose activity depends on their clusters. Organisms have developed machineries to promote the *in vivo* biogenesis of Fe-S clusters in part to protect cells from the deleterious effects of free Fe^2+^ and S^2–^ ions (5 to 9). Two types of *de novo* Fe-S cluster biogenesis machineries ISC (Iron Sulfur Cluster) and SUF (Sulfur mobilization) have been described in prokaryotes and eukaryotes. These two machineries operate using the same core principles. Briefly, a cysteine desulfurase mobilizes sulfur from l-cysteine, a scaffold protein provides a molecular platform allowing iron and sulfur to assemble and form a cluster, and a carrier protein delivers the cluster to the terminal apo-target (5 to 8). More recently, two minimal Fe-S cluster biogenesis machineries encoding ISC-like and SUF-like components have been described: MIS (minimal iron-sulfur) and SMS (suf minimal system), respectively (9).

As established in the model organism, Escherichia coli, maintaining Fe-S cluster homeostasis requires multiple layers of regulation, since making Fe-S clusters is absolutely required to ensure basic cellular functions. E. coli relies on both the ISC and SUF machineries that are encoded by the *iscRSUA-hscBA-fdx-iscX* and *sufABCDSE* operons, respectively. ISC is the housekeeping system, while the SUF system functions as a back-up system when ISC cannot satisfy the Fe-S cluster demands of cells. As revealed by genetic studies, Fe-S cluster homeostasis is ensured by controlling expression of the two machineries in E. coli at both the transcriptional and posttranscriptional levels (10, 11). At the transcriptional level, IscR, a member of the Rrf2 winged-helix-turn-helix family of transcriptional regulators, plays a major role in the control of both the *isc* and *suf* operons (12 to 15). Members of the Rrf2 superfamilly are highly versatile in sensing environmental inputs, such as iron limitation, oxidative stress, and/or nitrosative stress. Because these inputs are often at the heart of host–pathogen interactions, it is not surprising that Rrf2 members have been shown to be important for the virulence of several human pathogens, such as IscR of Yersinia tuberculosis and of Salmonella enterica (16, 17). IscR is itself a [2Fe-2S] cluster-containing protein encoded by the first gene of the operon encoding the ISC machinery (12). When loaded with a [2Fe-2S] cluster, holo-IscR recognizes a type 1 DNA-binding motif in the *isc* promoter to repress transcription, leading to a negative feedback loop (12, 14). When [2Fe-2S] cluster occupancy of IscR decreases, repression of the *isc* operon is alleviated and as a result IscR protein levels increase sufficiently to bind to a type 2 DNA-binding motif of the *suf* operon to activate transcription (13 to 15, 18, 19). Because maturation of IscR occurs preferentially by the ISC machinery, it possesses the remarkable capacity to sense when the ISC machinery is overwhelmed and to activate expression of the *suf* genes (10, 11, 20). The basal expression level of the *suf* operon is low, requiring activation by IscR to ensure growth of E. coli mutants relying solely on the SUF machinery (21). Enterobacteria possessing both ISC and SUF systems, such as Dickeya dadantii, exhibit the same IscR-dependent regulatory mechanism of the *isc* and *suf* operons as described for E. coli (22, 23). To ensure upregulation of the SUF machinery under stress conditions that are unfavorable for Fe-S cluster biogenesis, such as oxidative stress and iron restriction, *suf* expression is additionally controlled by the transcriptional regulators, OxyR and Fur, respectively (13, 18, 24, 25). Additionally, during iron limitation, posttranscriptional control via the small RNA RyhB acts negatively on *isc* expression (26).

Myxobacteria are Gram-negative soil-dwelling δ-proteobacteria that prey on pathogen and nonpathogen microorganisms (bacteria, yeast, fungi) using epibiotic strategies (27). Myxobacteria possess some of the largest genomes encountered in bacteria (~9 to 16 Mb), which was shown to encode an important reservoir of secondary metabolites. Interestingly, it has been shown that many biosynthetic pathways for secondary metabolites involve Fe-S-containing proteins for a core biosynthesis reaction *per se* or for modification of the metabolite itself (28). It is the case of myxovirescin, an antibiotic isolated from myxobacteria used against human pathogens, that has been shown to exhibit a novel mode of action (29, 30). These fascinating bacteria are also studied for their complex lifestyle involving multicellular development and social cooperative behavior (31 to 34). Myxobacteria undergo a biphasic life cycle depending on environmental cues, consisting of a vegetative phase and a complex developmental phase. Under vegetative conditions, cells secrete antibiotics, bioactive compounds, or degradative enzymes in amounts sufficient to immobilize and digest other bacteria (27, 35). When deprived of nutrients, most cells aggregate to form multicellular fruiting bodies, which support the development of metabolically dormant spherical myxospores (36). Myxococcus xanthus is one of the best studied members of *Myxobacteria*. As with most myxobacteria, M. xanthus is a strict aerobe and thus is very likely frequently exposed to reactive oxygen species (ROS). Because O_2_ decreases iron availability, M. xanthus may also experience iron limitation in its natural soil environment. In addition, in its natural habitat, M. xanthus encounters a large community of microorganisms producing oxidative compounds and iron chelators (37). These environmental factors are well documented to be adverse for the activity of Fe-S cluster proteins (38). Strikingly, how strict aerobes like M. xanthus maintain Fe-S cluster homeostasis has not been studied.

Here, we investigate how M. xanthus maintains and controls Fe-S cluster homeostasis, in comparison to the facultative anaerobe, E. coli. We show that M. xanthus possesses two Fe-S cluster biogenesis machineries, ISC and SUF. We report that M. xanthus uses a previously uncharacterized Rrf2-family member (MXAN_1152), renamed RisR here (Rrf2-type Iron Sulfur cluster homeostasis Regulator), to control expression of the two Fe-S cluster biogenesis pathways utilizing a genetic circuitry that suits its aerobic lifestyle. Indeed, we show that isolated RisR appears to harbor a Fe-S cluster and this holo-form acts as a repressor of both *isc* and *suf* operons. Maturation of RisR is directed by either the ISC or SUF machineries. We also show that the transcription upregulation of M. xanthus *isc* and *suf* operons occurs in response to oxidative stress or iron limitation and results solely from loss of RisR repression. Therefore, in M. xanthus the same RisR-dependent mechanism of repression controls the expression of both ISC and SUF machineries. The phenotypic characterization of Δ*iscU* and Δ*sufBCD* M. xanthus mutants revealed that, in contrast to E. coli, ISC and SUF appear to be two housekeeping machineries that are fully interchangeable to maintain appropriate levels of Fe-S cluster biogenesis. Nevertheless, as was found with E. coli, the SUF machinery plays a greater role than the ISC machinery in stress conditions. Altogether, our work reveals the function of a Rrf2-family regulator and a diverged transcriptional wiring that serves to directly coordinate the use of two Fe-S cluster biogenesis machineries to support aerobic growth of M. xanthus.

## RESULTS

### M. xanthus has two operons encoding the ISC and SUF machineries.

**FIG 1:**
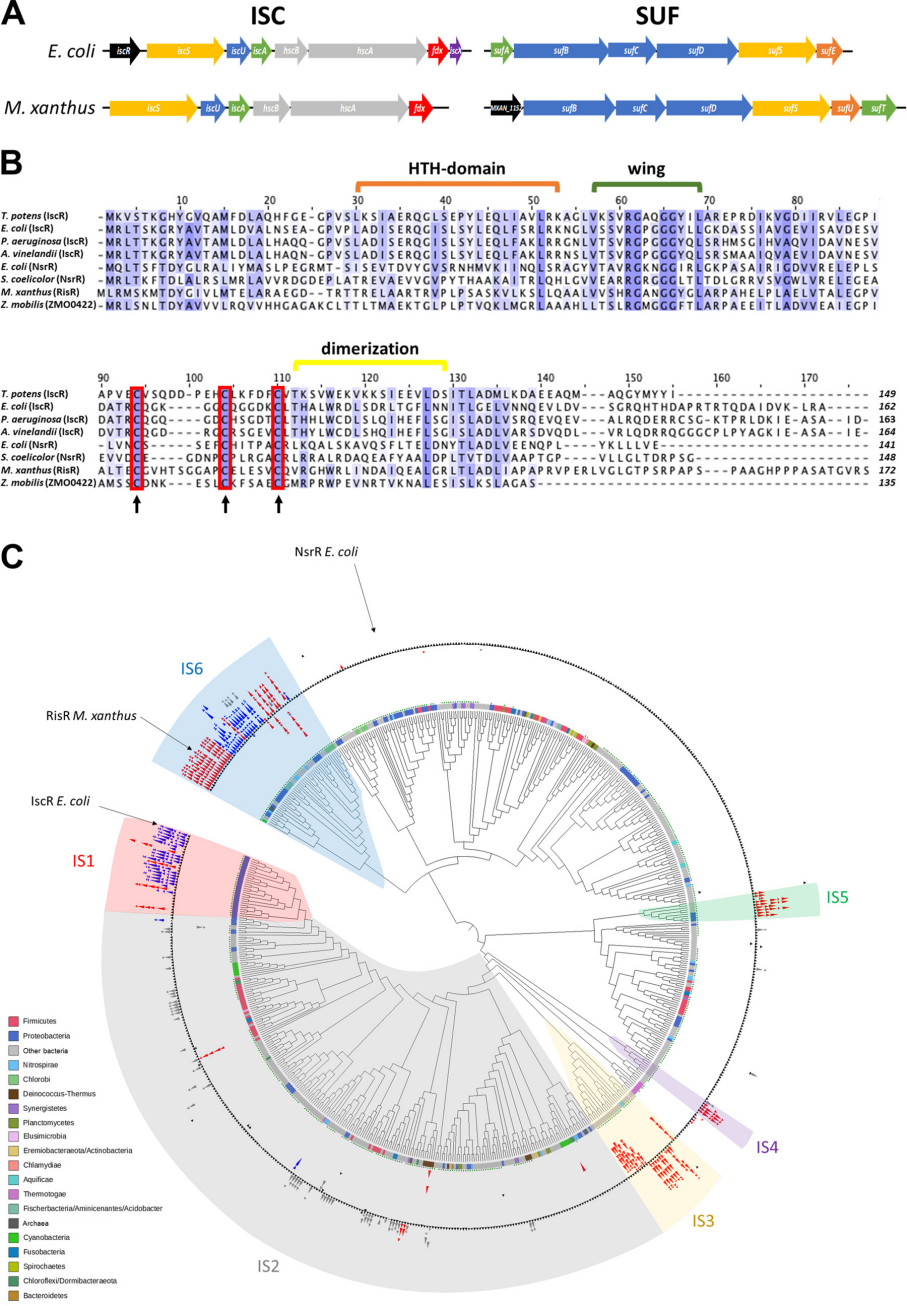
Identification of the Fe-S cluster biogenesis components in M. xanthus and phylogeny of Rrf2 family proteins. (A) Comparison of the genomic organization of the *isc* and *suf* gene clusters in E. coli and M. xanthus. Same color indicates gene function conservation, transcriptional regulator (black), cysteine desulfurase (yellow), sulfur acceptor (orange), scaffold (blue), Fe-S cluster carrier (green), chaperone/cochaperone, (gray), and electron donor (red). Purple indicates the *iscX* gene whose function in the Fe-S cluster biogenesis process remains to be characterized. (B) Multiple sequence alignment between M. xanthus RisR, IscR, and NrsR homologs from different bacterial species (Thermincola potens, E. coli, Pseudomonas aeruginosa, Azotobacter vinelandii, Streptomyces coelicolor, and Zymomonas mobilis). The protein sequences have been aligned using Clustal Omega, and BLOSUM 62 has been used for scoring alignments (threshold 25%). The red boxes represent the cysteine residues involved in the binding of the Fe-S cluster in E. coli IscR and conserved in M. xanthus RisR. Strictly conserved and partly conserved amino acids are highlighted in a blue color gradient from dark to light, respectively. Residues involved in IscR in wing and HTH-domain (green and orange brackets, respectively) and the dimerization helix (yellow bracket) are shown. (C) Cladogram of Rrf2 family proteins phylogeny. The circles at branches correspond to ultrafast bootstrap >95%. The strip around the tree indicates the corresponding major phyla at the bottom left side of the figure. The green dots indicate the presence of the three cysteines involved in the Fe-S cluster binding. Black triangles represent Rrf2 encoding genes, and the red, blue, and gray triangles indicate the SUF, ISC, and MIS machineries, respectively, at the *rrf2* vicinity. Major groups in synteny with Fe-S cluster machinery genes are indicated (IS1-6). IS, iron sulfur.

We first examined the M. xanthus genome for genes encoding Fe-S cluster biogenesis components. BLAST analysis revealed two gene clusters that encode proteins sharing sequence similarities with components of the E. coli ISC and SUF machineries (Fig. 1A and Fig. S1). A six-gene locus, from *MXAN_5001* to *MXAN_5006*, has a genomic organization similar to the E. coli *iscSUA-hscBA-fdx-iscX* gene locus (Fig. 1A), except for lacking *iscX* within the operon or elsewhere on the genome. Also, the M. xanthus *isc* locus does not contain a gene orthologous to E. coli *iscR*. However, a gene encoding an uncharacterized Rrf2 family member was found upstream of the *suf* operon, which is predicted to code for the SUF proteins, SufB, SufC, SufD, SufS, SufU, and SufT (*MXAN_1153* to *MXAN_1158*) (Fig. 1A and Fig. S1). This gene of unknown function, *MXAN_1152*, was renamed here as *risR* (Rrf2-type Iron Sulfur cluster homeostasis Regulator).

10.1128/mbio.03001-22.1 FIG S1 Homology of the ISC and SUF proteins of M. xanthus. Homology of the ISC and SUF proteins of M. xanthus was obtained using BLASTP from KEGG. M. xanthus query proteins are encoded by the genes indicated by an arrow. The percentage of homology (second row of each table) between the query protein and the protein of the specified organism (first and third row of each table) is shown. Same color indicates gene function conservation: cysteine desulfurase (yellow), sulfur acceptor (orange), scaffold (blue), Fe-S cluster carrier (green), chaperone/cochaperone, (grey), electron donor (red). Download FIG S1, TIF file, 0.5 MB. Copyright © 2023 Sourice et al. 2023 Sourice et al. https://creativecommons.org/licenses/by/4.0/ This content is distributed under the terms of the Creative Commons Attribution 4.0 International license.

Reverse transcription PCR (RT-PCR) analysis demonstrated that M. xanthus *iscS*, *iscU*, *iscA*, *hscB*, *hscA*, and *fdx* genes are organized in one transcriptional unit (Fig. S2A). Our data also revealed that the last gene of the M. xanthus *isc* operon is a gene encoding a tRNA^Leu^ (CAG). Using the same approach, we showed that *risR*, *sufB*, *sufC*, *sufD*, *sufS*, *sufU*, and *sufT* genes are organized in an operon (Fig. S2B). Altogether, our results show that M. xanthus possesses both the ISC and SUF Fe-S cluster biogenesis machineries, which are encoded by two operons: *iscSUA-hscBA-fdx-tRNA^Leu^* and *risR*-*sufBCDSUT*.

10.1128/mbio.03001-22.2 FIG S2 The *isc* and *suf* gene clusters are organized in operons in M. xanthus. (A) The *isc* gene cluster organization. PCR products of intergenic regions among (a) upstream_*isc*-*iscS,* (b) *iscS-iscU,* (c) *iscU-iscA,* (d) *iscA-hscB,* (e) *hscB-hscA,* (f) *hscA-fdx,* (g) *fdx-tRNA-Leu*, and (h) *tRNA-Leu-downstream_isc* from genomic DNA (lanes 1), RNA (lanes 2), and cDNA (lanes 3). Molecular size markers are indicated on the left of each panel (lanes M). Structural organization of the *isc* gene cluster is shown below. The arrow indicates the direction of transcription. The size of the boxes corresponds to the relative size of each gene. (B) The *suf* gene cluster organization. PCR products of intergenic regions between (a) upstream_*suf*-*risR,* (b) *risR-sufB,* (c) *sufB-sufC,* (d) *sufC-sufD,* (e) *sufD-sufS,* (f) *sufS-sufU,* (g) *sufU-sufT*, and (h) *sufT-downstream_suf* from genomic DNA (lanes 1), RNA (lanes 2), and cDNA (lanes 3). Molecular size markers are indicated on the left of each panel (lanes M). Organization of the *suf* gene cluster is shown below. The arrow indicates the direction of transcription. The size of the boxes corresponds to the relative size of each gene. Download FIG S2, TIF file, 0.8 MB. Copyright © 2023 Sourice et al. 2023 Sourice et al. https://creativecommons.org/licenses/by/4.0/ This content is distributed under the terms of the Creative Commons Attribution 4.0 International license.

### RisR defines a new subfamily of Rrf2-type regulators.

We asked how deeply RisR is distributed among prokaryotes and whether it is phylogenetically related to the well-characterized IscR from E. coli (EcIscR). Previous studies described the diversity of the Rrf2 protein family and highlighted several subfamilies (39, 40). However, the taxonomic sampling used was either restricted to *Alphaproteobacteria* or strongly biased in favor of *Proteobacteria* and *Firmicutes* given the sequences available at the time. Accordingly, we reinvestigated the Rrf2 protein family using an updated protein database representing the current known diversity of prokaryotes with a same-range number of representative organisms per phyla (a total of 553 proteomes) (Table S1). We identified 641 members of the Rrf2 family (Table S2). Five sequences corresponded to Archaea while 636 belonged to Bacteria (Table S2). The multiple sequence alignment showed that the majority of homologues, including RisR, had the three conserved cysteine residues associated with binding the [2Fe-2S] cluster in E. coli IscR (41) (Fig. 1B; and supplemental data available at https://figshare.com/articles/dataset/Sourice_et_al_Supplementary_data/21583104). Furthermore, we observed that the vast majority of the Rrf2 protein members possess the arginine residue (Arg59 in *Ec*IscR) (or a Lysine) whose side chain was shown in E. coli IscR to extend into the DNA minor groove (Fig. 1B and supplemental data), indicating conservation of the “wing” motif interaction with DNA (42).

10.1128/mbio.03001-22.7 TABLE S1 List of proteomes used in the phylogenetic analysis. Download Table S1, XLSX file, 0.5 MB. Copyright © 2023 Sourice et al. 2023 Sourice et al. https://creativecommons.org/licenses/by/4.0/ This content is distributed under the terms of the Creative Commons Attribution 4.0 International license.

10.1128/mbio.03001-22.8 TABLE S2 Taxonomic distribution of Rrf2 proteins in the 553 prokaryotes. Download Table S2, XLSX file, 0.05 MB. Copyright © 2023 Sourice et al. 2023 Sourice et al. https://creativecommons.org/licenses/by/4.0/ This content is distributed under the terms of the Creative Commons Attribution 4.0 International license.

From the alignment, we inferred a maximum likelihood phylogeny of the prokaryotic Rrf2 regulators (Fig. 1C). We also mapped the genomic context of each corresponding gene with respect to the genes involved in Fe-S biogenesis (e.g., cysteine desulfurases, U-type scaffolds, carriers) (Table S3). We mapped the genes of the SUF system (red), the ISC system (blue), and the newly described two-component MIS (minimal iron-sulfur) system (gray) (Fig. 1C) (43). Surprisingly, *rrf2* genes in the vicinity of Fe-S cluster biogenesis genes did not form a monophyletic group but instead were patchily distributed along the phylogeny (Fig. 1C). This suggests several independent recruitments of Rrf2-type regulators to the relevant Fe-S cluster biogenesis machinery during the evolution of prokaryotes. The mapping of the Fe-S cluster biogenesis system gene clusters on the Rrf2 phylogeny allowed us to delineate six subgroups of Rrf2 regulators possibly involved in Fe-S cluster biogenesis regulation (IS [iron-sulfur]1 to IS6) (Fig. 1C and Table S3). Strikingly, IscR from E. coli and RisR from M. xanthus belong to very distant groups, IS1 and IS6, respectively (Fig. 1C). IS1 is only represented by *Proteobacteria*, while IS6 includes a mix of unrelated phyla (i.e., *Chlorobi*, *Nitrospirae*, *Acidobacteria*, *Proteobacteria*, *Cyanobacteria*) (Fig. 1C ; Table S3). Moreover, IS1 and IS6 encompass a mix between SUF and ISC-associated Rrf2, in red and blue, respectively (Fig. 1C), indicating that Rrf2 likely switched several times from one Fe-S cluster biogenesis system to another. For example, Nitrosomonas europaea (IS1) and Chthonomonas calidirosea (IS6) Rrf2 likely switched from ISC (majority of the sister branches) to SUF genomic cluster within their respective IS. Altogether, these results suggest that E. coli IscR and M. xanthus RisR emerged independently through a very complex evolutionary history, involving potential functional shifts by switching between ISC and SUF systems and via several horizontal gene transfers (HGTs) especially for IS6. Hence, E. coli IscR and M. xanthus RisR belong to phylogenetically distinct groups. Interestingly, no member of the Rrf2 IS6 family has been investigated yet.

10.1128/mbio.03001-22.9 TABLE S3 Taxonomic distribution of Rrf2 subgroups from IS1 to IS6 and detailed synteny with Fe-S cluster biogenesis machineries. Download Table S3, XLSX file, 0.3 MB. Copyright © 2023 Sourice et al. 2023 Sourice et al. https://creativecommons.org/licenses/by/4.0/ This content is distributed under the terms of the Creative Commons Attribution 4.0 International license.

### Holo-RisR downregulates expression of both *isc* and *suf* operons *in* M. xanthus.

**FIG 2:**
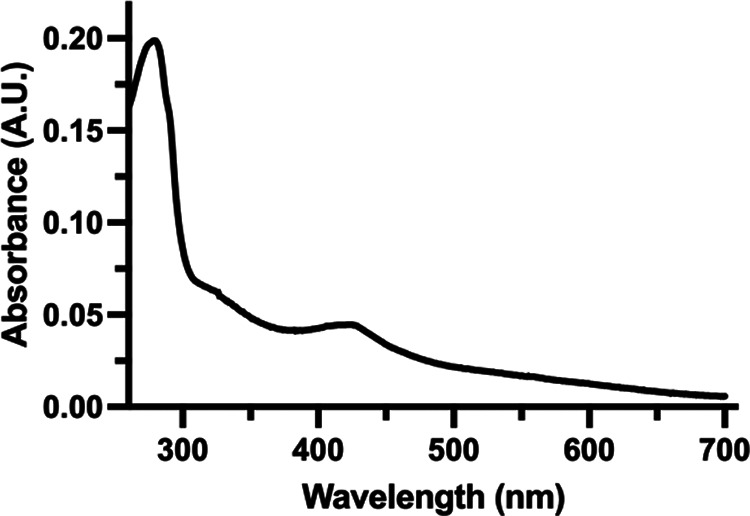
The UV-visible spectrum suggests that RisR binds a Fe-S cluster. UV-visible spectrum of anaerobically isolated N-terminal Strep-tag RisR protein (6.2 μM). The spectrum shows two characteristic peaks with maxima at ~330 nm and ~420 nm that might indicate the presence of [4Fe-4S] clusters.

To biochemically characterize RisR, a Strep-tagged variant was overproduced in E. coli. Isolation of RisR under aerobic conditions led to degradation of the cluster over time. Therefore, RisR was purified under anaerobic conditions, and all *in vitro* experiments were carried out in the absence of O_2_ to maintain cluster integrity. RisR was purified to homogeneity and displayed a green color with a visible absorption spectrum exhibiting two peaks around 330 nm and 420 nm, suggesting the presence of a [4Fe-4S] cluster (Fig. 2).

**FIG 3:**
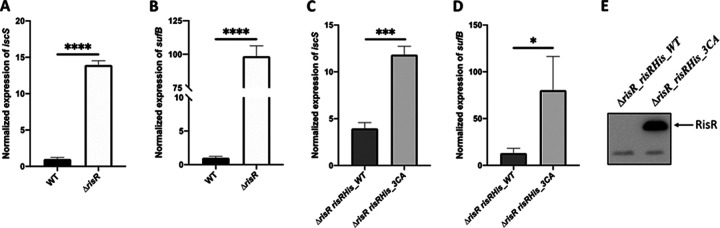
Holo-RisR downregulates the expression of both *isc* and *suf* operons. RT-qPCR analysis of the transcriptional levels of *isc* (A, C) and *suf* (B, D) operons in M. xanthus strains WT (panels A and B, black bars), *ΔrisR* (panels A and B, white bars), *ΔrisR risR_WT* (panels C and D, dark gray bars), and *ΔrisR risR_3CA* (panels C and D, light gray bars). The cultures used for RNA preparation were grown in CYE medium, and relative mRNA levels for each gene were normalized to 16S RNA levels. The amount of transcript in the WT strain is taken as reference. Error bars represent the standard deviation of three biological replicates. Statistical analysis was performed with Student’s test (panels A and B, ****, *P* < 0.0001; panel C, ***, *P* < 0.001; panel D, *, *P* < 0.05). Statistical analysis indicated that the difference between the expression levels of *isc*, or *suf*, in the *ΔrisR risRHis_3CA* mutant compared to *ΔrisR* was not significant. (E) Twenty-five micrograms of total protein extracts were analyzed for RisR protein levels by Western blot using antibodies raised against the histidine tag.

To test whether RisR controls the expression of the Fe-S cluster biogenesis machineries in M. xanthus, an in-frame deletion mutant lacking the *risR* gene (*MXAN_1152*) was constructed (*ΔrisR*), and expression of the *iscS* and *sufB* genes was monitored by qRT-PCR, as a read-out of the expression of *isc* and *suf* operons, respectively. In the presence of RisR, expression of the *isc* operon was 4-fold higher than the *suf* operon (Fig. S3). In the *ΔrisR* mutant, the transcriptional levels of *isc* and *suf* were ~15-fold and ~90-fold greater than in the M. xanthus wild-type (WT) strain, respectively, leading to the same order of magnitude of *isc* and *suf* transcript levels (Fig. 3A and B and Fig. S3). This indicates that RisR negatively controls the expression of both *isc* and *suf* operons.

10.1128/mbio.03001-22.3 FIG S3 Activities of the *suf* and *iscS* promoters in physiological conditions. Transcript amount of *iscS* (white bars) and *sufB* (black bars) genes in M. xanthus WT and Δ*risR* strains. The cultures used for RNA preparation were at OD_600nm_ 0.5 in CYE medium. Normalization was performed using the 16S RNA encoding gene. Error bars show the standard error of three biological replicates. Statistical analysis was performed with a *t* test (**, *P* < 0.01; ns, not significant). Download FIG S3, TIF file, 0.3 MB. Copyright © 2023 Sourice et al. 2023 Sourice et al. https://creativecommons.org/licenses/by/4.0/ This content is distributed under the terms of the Creative Commons Attribution 4.0 International license.

To investigate whether RisR requires coordination of the Fe-S cluster for its repressor function, the three predicted cluster-coordinating cysteine residues of RisR, Cys92, Cys102, and Cys108, were substituted to alanine, leading to the RisR_3CA variant. The ectopic *risR*_*3CA* allele, under the control of its own promoter, was inserted into the chromosome of the M. xanthus *ΔrisR* mutant at the phage Mx8 *attB* site, yielding the *ΔrisR risRHis_3CA* mutant. In this construct, the *suf* operon is still controlled by the native *suf* promoter. As the RisR_3CA variant also possesses an N-terminal His tag, the gene encoding N-terminal His-tagged WT RisR was similarly introduced at the phage Mx8 *attB* site in the *ΔrisR* mutant as a positive control, yielding the *ΔrisR risRHis_WT* strain. Fig. 3C and D showed that ectopically produced WT RisR still represses *isc* and *suf* expression, although repression was not as efficient as in the WT strain (Fig. 3). We further demonstrated that *isc* and *suf* gene expression was drastically upregulated in the *ΔrisR risRHis_3CA* mutant compared to the *ΔrisR risRHis_WT* strain (Fig. 3C and D). Expression levels of *isc* and *suf* in the *ΔrisR risRHis_3CA* mutant were similar in magnitude as the *ΔrisR* mutant (Fig. 3). Western blot experiments using anti-His tag antibodies indicated that the RisR_3CA variant was indeed produced at levels much greater than WT RisR as would be expected if holo-RisR negatively auto-regulates its own synthesis and WT RisR is not produced at a level sufficient to be detected by Western blot (Fig. 3E).

Altogether, these results indicate that the RisR_3CA variant lost the ability to downregulate *isc* and *suf* operons, suggesting that in M. xanthus, [4Fe-4S] holo-RisR downregulates expression of both the operons encoding ISC and SUF Fe-S cluster biogenesis machineries.

### Holo-RisR from M. xanthus binds to a dedicated motif upstream of the *isc* and *suf* operons.

**FIG 4:**
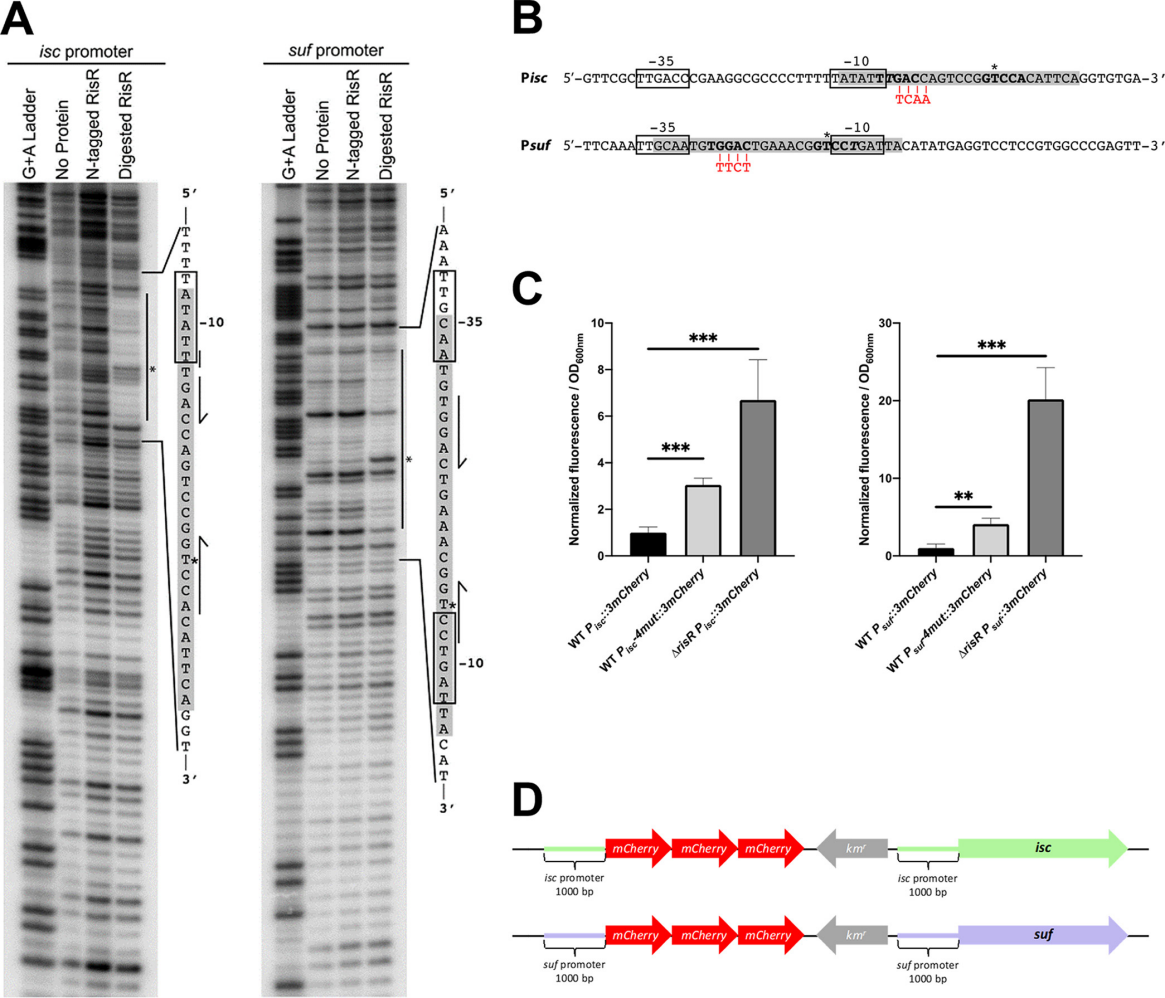
Holo-RisR binds to imperfect palindromic motifs in *isc* and *suf* promoters. (A) DNase I footprinting was used to identify the region where RisR binds within the *suf* and *isc* promoters from M. xanthus. RisR (1 μM) with or without the N-terminal StrepII tag was incubated with approximately 10 nM DNA prior to DNase I treatment. The vertical line indicates the protection area in each promoter. In the sequence, the gray box indicates the RisR protection region, and half arrows show bases corresponding to a palindromic motif present in both promoters. The predicted −10 and −35 hexamer RNA polymerase binding sites are boxed, and * shows a hypersensitive site evident after protein binding. (B) Sequence of RisR-binding motif identified in the *suf* and *isc* operon promoters from M. xanthus. The putative −10 and −35 hexamer RNA polymerase binding sites are boxed. The RisR-binding regions identified by DNase I footprinting are highlighted in gray, and in bold are shown bases corresponding to a palindromic motif present in both promoters. Mismatches in one of the half-sites are indicated in italic, and * shows hypersensitive sites. The nucleotides mutated by substitution are shown in red. (C) Relative fluorescence of the WT strain carrying the 3mCherry fused to the WT or mutated (4mut) promoters of *suf* or *isc* operons, and the Δ*risR* strain with the WT promoters fused to 3mCherry. Results represent the normalization of the fluorescence over the OD_600nm_. These data were normalized to the WT strain carrying 3mCherry fused to the WT promoter of *suf* or *isc* operons. Each point represents the mean from 6 experiments; error bars represent standard deviations. Statistical analysis was performed with a Dunnet test; **, *P* < 0.01; ***, *P* < 0.001. (D) Schemes of the chromosomal reporter fusions used are shown.

To test whether RisR binds directly to the *suf* and *isc* promoters and identify the location of the RisR binding sites, DNase I footprinting experiments were performed under anaerobic conditions (Fig. 4A). We observed a RisR-dependent DNase I protected region for both promoters of ~30 bp. For the *isc* promoter, RisR binds a segment of the promoter encompassing most of the predicted −10 hexamer and the putative transcription initiation start site for RNA polymerase (Fig. 4A). For the *suf* promoter, the protected region overlaps both the predicted −35 hexamer and −10 hexamer required for RNA polymerase binding (Fig. 4A). In both promoters, we observed the presence of a hypersensitive site within the protected regions. The N-terminal Strep-II tag fused to RisR for purification interferes with DNA binding, since protection from DNase I was only observed after treatment with enterokinase that removes the tag.

Within the RisR-protected regions of M. xanthus *isc* and *suf* operons is a conserved imperfect palindromic sequence 5′Tt/gGAC-N_7_ -GTCCt/a3′ flanked by AT-rich regions typical of proteins that use wing-helix-turn-helix motifs to recognize DNA in a site-specific manner (Fig. 4B). To test the importance of the palindromic sequence to RisR function, we disrupted the symmetry of one half-site by mutation in a region outside the −35 and −10 predicted hexamers and assayed whether RisR repression was compromised *in vivo* (Fig. 4B and C). The effect of the mutations on *suf* and *isc* expression was assayed using chromosomal *P_isc_::_3_mCherry* and *P_suf_::_3_mCherry* reporter gene fusions, and their corresponding palindromic site mutated versions, *P_isc_::4mut-_3_mCherry* and *P_suf_::4mut-_3_mCherry* (see scheme Fig. 4D). The reporter gene fusions contained the region encompassing 1,000 bp upstream of the translational start and three tandem copies of the gene encoding the fluorescent marker mCherry to achieve signal amplification. The fusions were inserted at the native locus by plasmid integration, keeping intact the gene structure of the *isc* and *suf* operons (see scheme Fig. 4D). Fluorescence was recorded from M. xanthus cultures grown in casitone-yeast extract (CYE) medium in the exponential phase. We found that, compared to *P_isc_::_3_mCherry* and *P_suf_::_3_mCherry*, expression of the mutated reporter gene fusions was increased by 3- and 4-fold, respectively; however, the effect of disruption of the palindromic sequence on expression of the reporter fusion was not as dramatic as what was observed in the *ΔrisR* mutant strain (Fig. 4C).

Altogether, these results defined a conserved RisR binding site upstream of the *isc* and *suf* operons and showed a common role of these sequences in the direct repression of *isc* and *suf* expression by RisR.

### Under normal growth conditions, ISC and SUF machineries of M. xanthus are fully interchangeable.

To study the contribution of ISC and SUF pathways, we constructed M. xanthus mutants lacking the scaffold component of one or the other machinery, *ΔiscU* and *ΔsufBCD*. We also repeatedly tried to obtain the *ΔiscU ΔsufBCD* mutant but without success, suggesting a requirement for Fe-S protein(s) in an essential biological process(es) of M. xanthus. In rich liquid medium, the *ΔiscU* and *ΔsufBCD* mutants exhibited identical growth parameters to those of the WT strain (doubling time of 4.2 ± 0.1 h) (Fig. S4A). By spotting each strain on solid media and measuring flare expansion, we showed that absence of ISC or SUF machinery did not affect strain motility (Fig. S4B).

10.1128/mbio.03001-22.4 FIG S4 Growth, motility, and RisR maturation are not affected in strains lacking ISC or SUF machinery. (A) Growth curves of M. xanthus WT, Δ*iscU*, and Δ*sufBCD* strains with respective doubling times of 4.2, 4.8, and 4.5 h. Cultures were grown in CYE medium and standardized at OD_600nm_ 0.1 (t_0_). (B) Morphology and expansion of M. xanthus colonies from the WT, Δ*iscU*, and Δ*sufBCD* strains were observed on CYE medium plates containing 0.5% of agar after 48 h of growth at 32°C. The colony expansion of WT, Δ*iscU*, and Δ*sufBCD* strains at 48 h is shown as a bar (right side of each photograph). The colony expansion is calculated by subtracting the t_0_ spot diameter from the diameter of the colony at 48 h (t_f_) and dividing by 2 (scheme). The diameter was measured using Fidji software. The colony expansion is expressed in arbitrary units; error bars show the standard error of three biological replicates. Statistical analysis was performed with a Dunnet test (ns, not significant). (C) Relative expression of the *iscS* gene in M. xanthus WT (black bar), Δ*risR* (white bar), Δ*iscU* (light grey bar), and Δ*sufBCD* (dark grey bar) strains. The cultures used for RNA preparation were grown on CYE, and relative mRNA levels for each gene were normalized to 16S RNA levels. The amount of transcript in the WT strain is taken as reference. Error bars show the standard error of three biological replicates. Statistical analysis was performed using the Tukey test (****, *P* < 0.0001). Download FIG S4, TIF file, 1.3 MB. Copyright © 2023 Sourice et al. 2023 Sourice et al. https://creativecommons.org/licenses/by/4.0/ This content is distributed under the terms of the Creative Commons Attribution 4.0 International license.

We then investigated the participation of ISC and SUF pathways to homeostatic regulation, by analyzing their ability to maturate RisR. As a read-out for RisR maturation, we quantified by qRT-PCR the ability of RisR to repress the P*isc* promoter. We showed that the repressor activity of RisR was modestly diminished in either *ΔiscU* or *ΔsufBCD* mutants compared to the WT strain (Fig. S4C). This is contrast to E. coli IscR, for which we previously showed that maturation of IscR is drastically affected in the *ΔiscU* mutant, whereas the *ΔsufBCD* mutant retains the holoform of IscR (12, 20).

**FIG 5:**
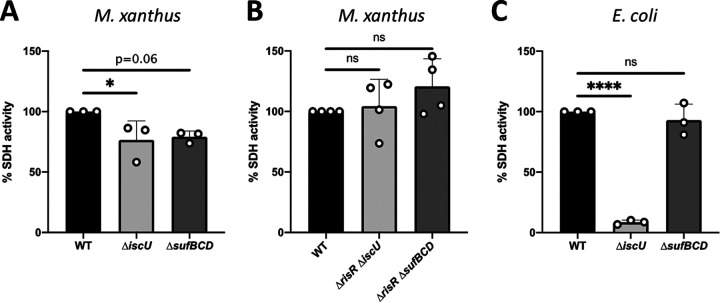
SDH activity is not affected in strains lacking ISC or SUF machinery. (A) SDH activity was determined by measuring polarographically the O_2_ consumption using succinate as the substrate from membranes of the M. xanthus WT (black bar), Δ*iscU* (light gray bar), and Δ*sufBCD* (dark gray bar) strains. Bar graphs show the percentage of O_2_ consumption relative to the WT set to 100%. Error bars show the standard error of three biological replicates. Statistical analysis was performed using the Dunnet test (*, *P* < 0.05). (B) SDH activities were determined by measuring the O_2_ consumption from the M. xanthus WT (black), *ΔrisR ΔiscU* (light gray bar), and *ΔrisR ΔsufBCD* (dark gray bar) membranes using succinate as electron donor. Bar graphs show the percentage of O_2_ consumption relative to the WT set to 100%. Error bars show the standard error of four biological replicates. Statistical analysis was performed using the Dunnet test (ns, not significant). (C) SDH activities were determined by measuring the O_2_ consumption from the E. coli WT (black bar), Δ*iscU* (light gray bar), and Δ*sufBCD* (dark gray bar) membranes using succinate as electron donor. Bar graphs show the percentage of O_2_ consumption relative to the WT set to 100%. Error bars show the standard error of three biological replicates. Statistical analysis was performed using the Dunnet test (****, *P* < 0.0001; ns, not significant).

To further explore the functional redundancy of ISC and SUF biogenesis pathways in M. xanthus, we assayed, *in vivo*, the activity of succinate dehydrogenase (SDH) using succinate as the substrate and O_2_ as the terminal electron acceptor. We chose SDH to assay since our analysis of the M. xanthus genome predicted that it contains genes coding for such Fe-S dependent activity (*MXAN_3539* and *MXAN_3540*). Membrane extracts prepared from the M. xanthus *ΔiscU* and *ΔsufBCD* mutants exhibited the same level of SDH activity, which was decreased by 20% compared to the M. xanthus WT strain (Fig. 5A). As a control, we used the same method to assay SDH activity in E. coli and the corresponding E. coli *ΔiscU* and *ΔsufBCD* mutants (Fig. 5C). Consistent with published data using other methods to measure SDH activity, we showed a drastic loss of SDH activity in the E. coli *ΔiscU* mutant, while the *ΔsufBCD* mutant exhibited WT level of SDH activity (Fig. 5C). In order to test whether alleviation of the RisR repression on Fe-S cluster biosynthesis machineries is sufficient to restore WT to the level of SDH activity in the *ΔsufBCD* and *ΔiscU* mutants, we constructed the *ΔrisR ΔsufBCD* and *ΔrisR ΔiscU* strains. Figure 5B showed the same measured activity in WT, *ΔrisR ΔsufBCD*, and *ΔrisR ΔiscU*, suggesting that increased amounts of either ISC or SUF permit WT level of SDH maturation.

Our data unveiled that in contrast to E. coli, M. xanthus ISC and SUF machineries support maturation of Fe-S proteins at the same level, as shown for RisR and the succinate-O_2_ reductase respiratory chain, suggesting that without stress, ISC and SUF can be fully interchangeable, but both are nevertheless required for maximal activity level of the WT strain.

### Up-regulation of M. xanthus *isc* and *suf* operons in stress conditions depends only on RisR.

**FIG 6:**
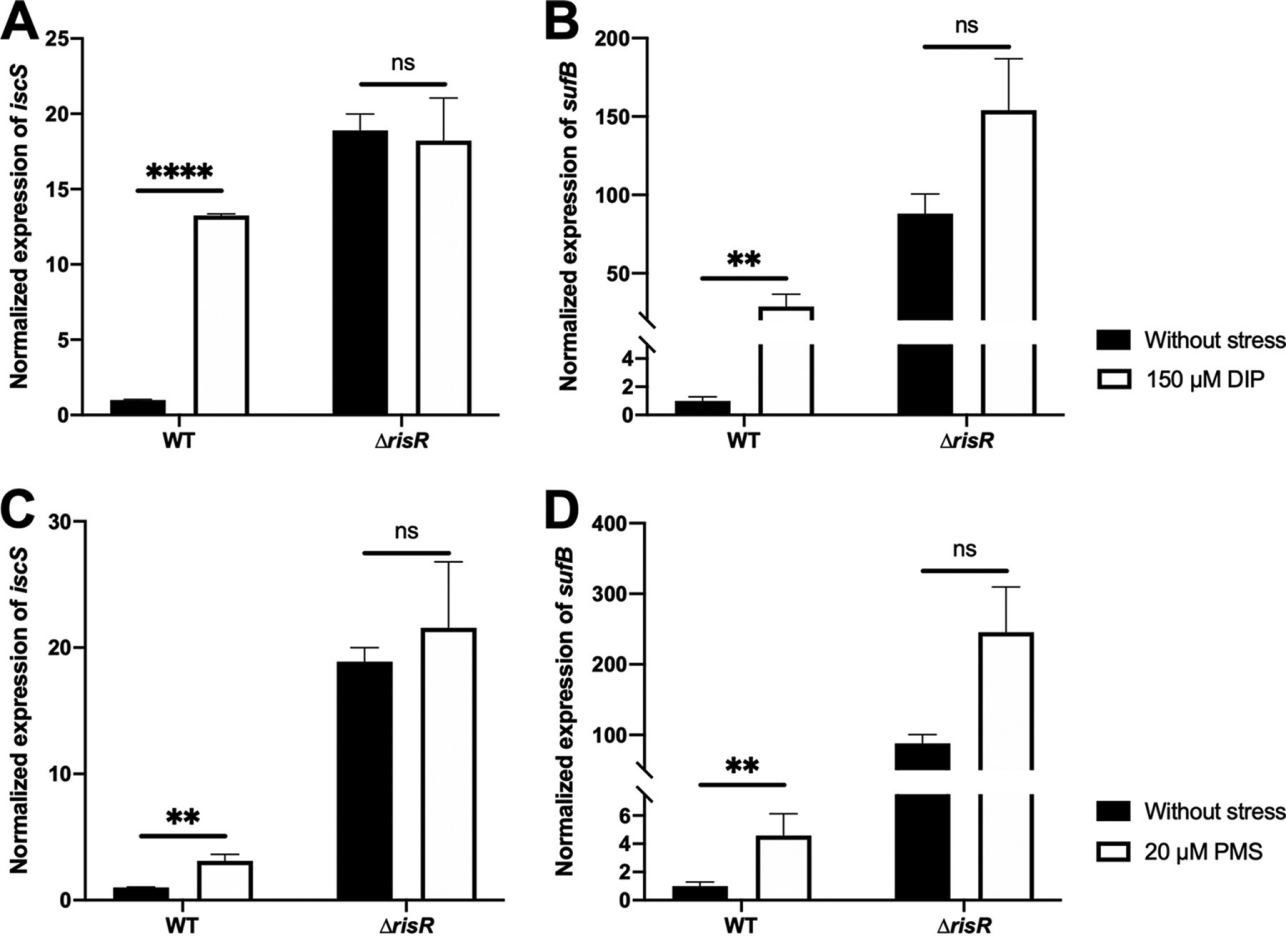
Activation of *isc* and *suf* operons during iron starvation and under oxidative stress depends only on RisR. Relative expression of (A) *isc* and (B) *suf* operons in M. xanthus WT and Δ*risR* cells untreated (black bars) or treated with DIP (white bars) strains. The cultures used for RNA preparation were grown from OD_600nm_ 0.2 to 0.5 on CYE containing or not 150 μM DIP. The relative mRNA levels for each gene were normalized to 16S RNA levels. The amount of transcript in the WT strain in the absence of stress is taken as reference. Error bars show the standard error of three biological replicates. Statistical analysis was performed with a Sidak test (**, *P* < 0.01; ****, *P* < 0.0001; ns, not significant). Relative expression of (C) *isc* and (D) *suf* operons in M. xanthus WT and Δ*risR* cells untreated (black bars) or treated with PMS (white bars) strains. The cultures used for RNA preparation were grown until OD_600nm_ 0.5 on CYE supplemented or not with 20 μM PMS. The amount of transcript in the WT strain grown in the absence of stress is taken as reference. Error bars show the standard error of three biological replicates. Statistical analysis was performed with a Sidak test (**, *P* < 0.01; ns, not significant).

It is well known that iron starvation and oxidative stress affect Fe-S cluster biogenesis and that cells shaped genetic controls to adapt (44, 45). To investigate the effect of iron starvation and oxidative stress on *isc* and *suf* operon expression in M. xanthus, cell cultures were treated with an iron chelator, 2,2′-dipyridyl (DIP) (150 μM), and a superoxide generator, phenazine-methosulfate (PMS) (20 μM). These concentrations of DIP and PMS were chosen because compared to the untreated culture, they were causing an increased doubling time of M. xanthus WT strain by ~2- and 2.5-fold, respectively (Fig. S5). Correspondingly, expression of the *isc* and *suf* operons, assessed by qRT-PCR, was also increased (Fig. 6); when the WT strain was cultured in the presence of DIP, *isc* and *suf* expression was upregulated by ~13- and 30-fold, respectively (Fig. 6A and B). Upon addition of PMS, *isc* and *suf* expression increased ~5- and 7-fold, respectively (Fig. 6C and D). No further derepression of *isc* and *suf* expression was observed in the *ΔrisR* mutant when cells were treated with DIP or PMS (Fig. 6), indicating that RisR is inactivated by DIP and PMS.

10.1128/mbio.03001-22.5 FIG S5 Growth curves of M. xanthus WT, Δ*iscU*, and Δ*sufBCD* strains in stress conditions. Strains were grown in CYE medium supplemented or not with 10 μM of PMS (right panel) and 150 μM of DIP (left panel). The dilution used to start the culture (T_0_) was done in order to normalize the OD_600nm_ to 0.1. Error bars show the standard error of two biological replicates. Download FIG S5, TIF file, 0.3 MB. Copyright © 2023 Sourice et al. 2023 Sourice et al. https://creativecommons.org/licenses/by/4.0/ This content is distributed under the terms of the Creative Commons Attribution 4.0 International license.

Taken together, we conclude that iron starvation and oxidative stress induce expression of both *isc* and *suf* through a RisR-dependent mechanism.

### SUF is required to sustain growth and to mature Fe-S proteins in iron starvation and under oxidative stress.

**FIG 7:**
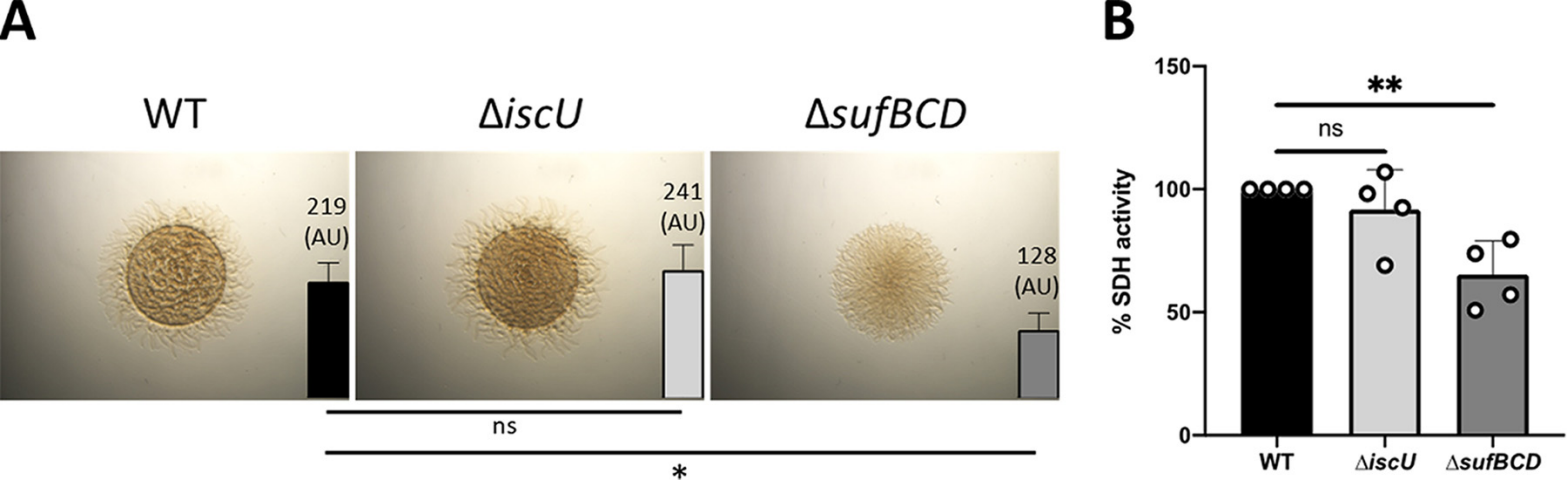
The SUF machinery is required during iron starvation for M. xanthus growth and motility. (A) Colony morphology and motility of the WT, Δ*iscU*, and Δ*sufBCD* strains in the presence of 100 μM DIP. Pictures of the colonies of M. xanthus WT, Δ*iscU*, and Δ*sufBCD* strains were taken after 48 h of growth on CYE plates with 0.5% of agar and 100 μM DIP. The colony expansion of WT, Δ*iscU*, and Δ*sufBCD* strains at 48 h is shown as a bar (right side of each photograph). The colony expansion is calculated by subtracting the t_0_ spot diameter from the diameter of the colony at 48 h (t_f_) and dividing by 2 (scheme, Fig. S4B). The colony expansion is expressed in arbitrary units, and error bars show the standard error of three biological replicates. Statistical analysis was performed with a Dunnet test (*, *P* < 0.05; ns, not significant). (B) SDH activity was determined by measuring the O_2_ consumption from membranes of the M. xanthus WT (black bar), Δ*iscU* (light gray bar), and Δ*sufBCD* (dark gray bar) using succinate as the substrate. Membranes were prepared from cultures grown in CYE medium in the presence of 100 μM DIP at OD_600nm_ between 0.4 and 0.6. Bar graphs show the percentage of O_2_ consumption relative to the WT set to 100%. Statistical analysis was performed with a Dunnet test (**, *P* < 0.01; ns, not significant).

We then assessed the relative importance of M. xanthus ISC and SUF machineries under conditions of iron starvation (DIP) or oxidative stress (PMS). In the presence of DIP, we showed that growth on rich liquid medium of the *ΔsufBCD* strain was significantly slower (doubling time of 11.8 ± 1.1 h) than for the WT and *ΔiscU* strains (doubling time of 8 ± 1.3 h and 7.2 ± 0.9 h, respectively) (Fig. S5). When grown on DIP-containing solid 0.5% agar, which is favorable for type IV pili motility (46), the WT and *ΔiscU* strains formed typical long flares, whereas the *ΔsufBCD* strain exhibited significantly reduced spot expansion growth (40% lower) and shorter flares (Fig. 7A). We also showed that the SDH activity in the *ΔsufBCD* strain treated with DIP was 43% lower than in the WT and *ΔiscU* strains (Fig. 7B).

The same experiments were performed with WT, *ΔiscU*, and *ΔsufBCD* strains under oxidative stress conditions. In the presence of PMS, the *ΔsufBCD* mutant exhibited growth, type IV motility, and SDH activity that were decreased compared to the WT strain (Fig. S6). In contrast, the *ΔiscU* mutant was indistinguishable from the WT strain (Fig. S6).

10.1128/mbio.03001-22.6 FIG S6 The SUF machinery is required during oxidative stress for M. xanthus growth and type IV motility. (A) Pictures of M. xanthus WT, Δ*iscU*, and Δ*sufBCD* colonies grown 48 h on CYE 0.5% agar plates with 10 μM of PMS. The colony expansion of WT, Δ*iscU*, and Δ*sufBCD* strains at 48 h is shown as a bar (right side of each photograph). The colony expansion is calculated by subtracting the t_0_ spot diameter from the diameter of the colony at 48 h (t_f_) and dividing by 2 (scheme Fig. S4B). The colony expansion is expressed in arbitrary units; error bars show the standard error of three biological replicates. Statistical analysis was performed with a Dunnet test (**, *P* < 0.01; ns, not significant). (B) Succinate-oxygen reductase activity was determined by measuring the O_2_ consumption from membranes of the M. xanthus WT (black bar), Δ*iscU* (light grey bar), and Δ*sufBCD* (dark grey bar) using succinate as a substrate. Membranes were prepared from cultures grown in CYE medium in the presence of 10 μM of PMS at OD_600nm_ between 0.4 and 0.6. Bar graphs show the percentage of O_2_ consumption relative to the WT set to 100%. Error bars show the standard error of three biological replicates. Statistical analysis was performed using the Dunnet test (*, *P* < 0.05; **, *P* < 0.01; ns, not significant). Download FIG S6, TIF file, 0.9 MB. Copyright © 2023 Sourice et al. 2023 Sourice et al. https://creativecommons.org/licenses/by/4.0/ This content is distributed under the terms of the Creative Commons Attribution 4.0 International license.

Collectively, these results indicate that in *M. xanthus* the SUF machinery plays a more prominent role in Fe-S cluster biogenesis during iron starvation and under oxidative stress.

## DISCUSSION

In all organisms, maintaining Fe-S cluster homeostasis is an essential function that is finely controlled in order to sustain the Fe-S cluster demand without depriving iron from other iron-dependent processes. In addition, Fe-S cluster demand can also reflect environmental conditions, since the reactivity of some Fe-S clusters to agents such as O_2_ and ROS comes with a cost of cluster loss. Here, we provide the first example of how a bacterium, from outside the *Gammaproteobacteria* branch, controls two Fe-S cluster biogenesis machineries, ISC and SUF. The model bacteria used, M. xanthus, belongs to the δ-*Proteobacteria* branch and has attracted considerable research interest due to its ecological importance, multicellular development, and metabolite production, but for which nothing was known regarding Fe-S cluster homeostasis, despite that its genome encodes more than 90 predicted Fe-S proteins.

**FIG 8:**
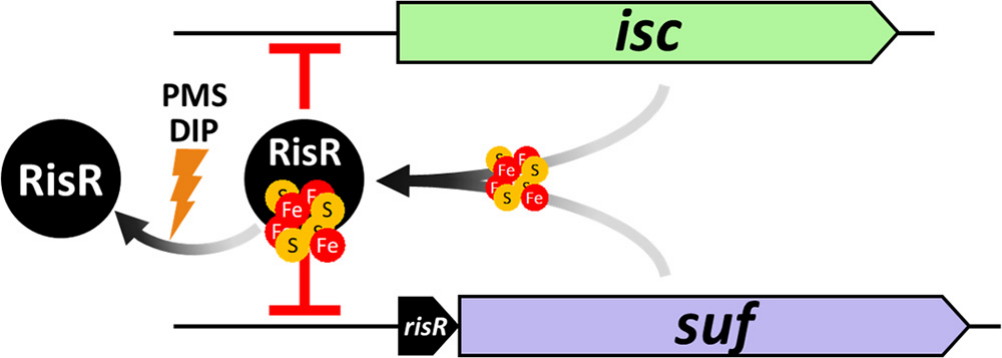
Model of regulation of *isc* and *suf* operons by RisR in M. xanthus. Under optimal growth conditions, both ISC and SUF can mature RisR. The [4Fe-4S]-RisR (holo-form) acts as a repressor of both operons. This repression can be removed in stress conditions, such as iron starvation or oxidative stress. These stress conditions lead to a lack of or an oxidation of the iron atoms of the RisR Fe-S cluster, presumably converting RisR to its apo-form. Under its apo-form, RisR cannot bind to the promoters of *isc* and *suf*; thus, both operons are upregulated.

Our results show that, similar to E. coli, M. xanthus relies on two Fe-S cluster machineries, ISC and SUF. Importantly, this study reveals that M. xanthus orchestrates the use of these two machineries via a genetic circuitry that is much simpler than that of *Enterobacteria* (7, 10, 11). Indeed, we found that M. xanthus uses a transcriptional regulator, RisR, that belongs to a previously uncharacterized group of the Rrf2-type proteins (40). RisR is a member of a subgroup that is phylogenetically most distant from that of E. coli IscR. From complementary *in vivo* and *in vitro* approaches, we established that control by RisR of Fe-S cluster homeostasis in M. xanthus is achieved by a negative feedback loop of both *isc* and *suf* operon expression (Fig. 8). In addition, Fe-S cluster biogenesis regulation in M. xanthus relies on a different transcriptional wiring than in E. coli, which allows M. xanthus SUF machinery, together with ISC, to fully participate in Fe-S cluster homeostatic control under normal conditions. A change in transcriptional wiring is also illustrated by the fact that the combination of *ΔrisR* and *ΔiscU* mutations in M. xanthus is viable, whereas the equivalent combination of *ΔiscR* and *ΔiscU* in E. coli is not (21). This study highlights the diversity and common themes that have emerged in phylogenetically distant bacteria to appropriately adjust Fe-S cluster homeostasis in response to environmental changes.

The phylogenetic analysis suggests that the Rrf2 family of transcriptional regulators went through a complex evolutionary history and has been recruited several times independently into the Fe-S cluster biosynthesis genomic clusters. IscR and RisR, of IS1 and IS6 groups, respectively, are two examples of such recruitment that is very likely explained by the presence of conserved cysteine residues allowing them to bind a Fe-S cluster, which permit a direct sensing of the Fe-S cluster status of the cell. Whether the Rrf2-type regulators, of groups IS2, IS3, IS4, and IS5, which are found in the vicinity of SUF-encoding genes or the newly described Fe-S cluster biogenesis MIS system, are Fe-S-binding proteins remains to be experimentally validated (43). However, the presence of three conserved cysteines strongly suggests that it is the case. For example, in *Synechocystis* sp. PCC 6803 and Mycobacterium tuberculosis, another type of Fe-S cluster-binding transcriptional regulator possessing an ArsR-like helix-turn-helix domain has also been recruited by the SUF machinery likely for the same reason (47 to 49).

Using a protein database representing the current known diversity of prokaryotes with a same-range number of representative organisms per phyla allowed us to pinpoint that in nearly half of the bacteria possessing at least one Rrf2 member, the gene is located in the vicinity of genes encoding Fe-S cluster biogenesis components. This supports the view that cells make wide usage of Rrf2-type regulators to adjust Fe-S cluster supply, suggesting that Rrf2 family members provide a flexible platform for regulating activity by Fe-S clusters. In addition, Rrf2-type regulators can also regulate other metabolic pathways, as shown in E. coli, Bacillus subtilis, and Rhizobium leguminosarum, which employ NsrR (IS5), CymR (IS2), and RirA for regulation of nitric oxide detoxification and cysteine and iron metabolism, respectively (50 to 52). More recently, SifR, an Rrf2-type regulator from Streptococcus pneumoniae, was shown to be a quinone sensor (53). Altogether, the Rrf2 family members can function broadly in gene regulation.

UV-visible spectroscopy showed that anaerobically purified RisR does bind an Fe-S cluster. Coordination of the cluster likely uses the three highly conserved cysteine residues (C92, C102, C108) as ligands, similar to other Fe-S binding members of the Rrf2 family such as *Ec*IscR and *Ec*NsrR (42, 54). Of significance, RisR is phylogenetically more closely related to E. coli [4Fe-4S] NsrR than to [2Fe-2S] IscR, and our visible absorption spectrum suggests that RisR would also bind a [4Fe-4S] cluster (41, 54). Future studies will aim to determine whether [4Fe-4S] versus [2Fe-2S] clusters might impose some differences in sensing Fe-S cluster cellular demand, in particular under conditions known to endanger cluster stability, such as iron starvation and oxidative stress. Interestingly, the most divergent region between RisR and the structurally determined Rrf2 regulators *Ec*IscR and *Ec*NsrR encompasses part of the putative Fe-S cluster-binding region and the dimerization helix just downstream of the Fe-S cluster-binding region. Thus, this study illustrates further the plasticity of Rrf2-type regulators, which by changing the residues surrounding the Fe-S cluster binding region might be able to diversify the type of Fe-S cluster and the environmental change perceived by the Fe-S cluster.

Direct binding of holo-RisR to the promoter region of M. xanthus *isc* and *suf* operons was shown by footprinting. Interestingly, the RisR DNA binding regions exhibit a core palindromic motif (GAC-N_7_-GTC, flanked by AT-rich regions), which is typical of proteins that use wing-helix-turn-helix motifs to bind DNA sites as homodimers. Indeed, the predicted DNA binding domain of RisR exhibits homology to the wing-helix-turn-helix domain of other Rrf2 regulators, especially the β-hairpin region that has been shown to insert into the minor groove (42, 54–57). Accordingly, the predicted β-hairpin region of RisR possesses the three Gly residues of the conserved Gx_2_GGx_2_L motif and the Arg59 residue, which directly interacts with DNA in the *Ec*IscR/*hyaA* and Streptomyces coelicolor (*Sc*) NsrR/*hmpA1* complex structure (42, 57). In contrast, the residues involved in base-specific recognition within the major groove, such as those determined in *Ec*IscR (Gln44, Ser40, Glu43), are not conserved in RisR. This is what one can expect from regulators that have evolved independently and whose specificity relies on a combination of specific amino acids and nucleotides. The repressor activity of RisR is likely to occur through occlusion of the RNA polymerase binding, since the putative RisR binding sites overlap the −10 and −35 hexamers upstream of the *suf* operon and the −10 hexamer upstream of the *isc* operon. The stronger repression of the *suf* genes compared to *isc* might be due to such differences in the location of the RisR-binding site.

Our model is that conditions that endanger Fe-S cluster homeostasis, such as the presence of a superoxide generator or an iron chelator, favor the apo-RisR form that we expect does not bind to DNA, leading to derepression of both the *isc* and *suf* operons (Fig. 8). This is consistent with the inability of the variant of RisR that is locked in the apo form (RisR_3CA) to exert its repressive activity. What prevents apo-RisR from binding to the *isc* and *suf* promoters remains to be determined. Hence, the loss of the cluster might introduce radical structural differences in the DNA-interacting region, such as in *Sc*NsrR, or more subtle difference such as in *Ec*IscR (42).

As found for most living organisms in which it has been investigated, our results suggest that Fe-S cluster biogenesis is very likely an essential process in M. xanthus. Indeed, we were unable to obtain the double Δ*iscU* Δ*sufBCD* mutant, whatever the conditions used to construct and select the second recombination event. Nevertheless, characterization of M. xanthus single *isc* and *suf* mutants revealed a striking difference from E. coli, which has been the paradigm for studies of bacteria having both the ISC and SUF Fe-S cluster biogenesis machineries. A surprising result in M. xanthus was the fact that in the absence of stress, cells expressing only one machinery, ISC or SUF, exhibited identical growth parameters and SDH activity. These results suggest that ISC and SUF are fully interchangeable in normal growth conditions. This is in contrast to E. coli, in which cells expressing only the SUF machinery show drastic growth defects and strong decreases in Fe-S protein maturation compared to cells expressing only the ISC system (58). The rationale for this difference is thought to result in part from the different wiring of *isc* and *suf* expression in these two organisms. The physiological consequence of expression of both machineries is that RisR can acquire its cluster from both the ISC and SUF machineries. Thus, in standard aerobic growth conditions, in contrast to E. coli, M. xanthus can use the SUF machinery to control Fe-S cluster homeostasis. Whether this reflects an adaptation associated with M. xanthus being a strict aerobe, and possibly being confronted with permanent endogenous oxidative stress and limited iron availability, is an attractive hypothesis.

In the presence of oxidative stress, and also during iron limitation, the Δ*sufBCD* mutant of M. xanthus exhibited growth defect and decreased SDH activity compared to the WT strain, indicating that the SUF machinery, as in E. coli, was required to cope with stress conditions that endanger Fe-S cluster biogenesis. Under the same conditions, the Δ*iscU* mutant of M. xanthus did not show any phenotypes. Altogether, these results are consistent with the view that, as for E. coli SUF proteins, the M. xanthus SUF proteins possess intrinsic biochemical properties, which allow them to outperform the ISC proteins in the face of iron starvation and oxidative stress. For example, the [2Fe-2S] cluster of the E. coli SufB scaffold protein was shown to be more stable than the E. coli IscU [2Fe-2S] cluster in the presence of hydrogen peroxide, oxygen, and an iron chelator (59). In the same vein, the E. coli SufS-SufE sulfur transfer pathway is more resistant to oxidative stress than IscS-IscU (60).

Furthermore, we show that even though the use of SUF during stress conditions resembles that observed in *Gammaproteobacteria* possessing both ISC and SUF such as E. coli, the regulatory strategy controlling *suf* expression in stress conditions diverges in M. xanthus (10, 21, 38, 61). Hence, in oxidative stress and iron starvation M. xanthus *suf* expression is controlled solely *via* derepression of RisR, while in E. coli, triggering expression of *suf* requires activation via IscR together with the action of dedicated stress sensors, OxyR in oxidative stress, and Fur in iron limitation.

Recent transcriptomic data have suggested upregulation of the *isc* and *suf* operons during the developmental cycle of M. xanthus, which was triggered upon nutrient starvation (62). Whether such upregulation is RisR dependent remains to be investigated; if so, this might reflect an increase in the Fe-S cluster demand during the progression of the multicellular developmental program of M. xanthus. Interestingly, in the ecological habitat of M. xanthus, upregulation of the *isc* and *suf* operons might occur, depending on the prey microorganism present. Hence, when M. xanthus is cocultured with Streptomyces coelicolor, both the *isc* and *suf* operons are upregulated, while when M. xanthus is in contact with S. meliloti, none of the genes of the *isc* and *suf* operons are found to be significantly differentially regulated (63, 64). Thus, future studies will aim to investigate the contribution of the of Fe-S cluster biogenesis machineries, and their genetic control, in the M. xanthus life cycle and in its interspecies interaction.

In summary, we describe here a new and different strategy deployed by the deltaproteobacterium M. xanthus to manage Fe-S cluster homeostasis using two machineries. In the vast majority of Deltaproteobacteria, SUF is the sole system present in these organisms. Whether the ISC machinery was horizontally acquired by M. xanthus or massively lost by Deltaproteobacteria except M. xanthus remains to be determined. Nevertheless, the presence of a tRNA at the 3′ extremity of the *isc* operon, which is known to be frequently used as the insertion site for foreign genetic materials, might favor the first scenario. The present study expands our knowledge on Fe-S cluster biogenesis control in bacteria exhibiting a complex social multicellular life cycle that succeeds in an Fe-S-cluster unfriendly aerobic environment. These results further highlight the constant molecular tinkering used by bacteria to adapt to their niche.

Finally, in the context of antimicrobial resistance, there is a tremendous need for new drugs. Genome mining of Myxobacteria has recently led to some excitement due to the prolific number of putative secondary metabolites, which in this regard are similar to actinomycetes and certain fungi (65). In parallel, the use of M. xanthus as a chassis for secondary metabolite production has recently attracted a lot of interest (29). Given the fact that Fe-S proteins have emerged as key enzymes in metabolite production/modification (28), our study paves the way to improve industrial-scale biosynthesis of Fe-S-cluster-dependent molecules in M. xanthus.

## MATERIALS AND METHODS

### Bacterial strains and growth conditions.

Strains used in this study are listed in Table S4. M. xanthus strains were grown at 32°C on casitone-yeast extract (CYE) agar plates or in liquid medium on a rotary shaker at 160 rpm. Plates contained 0.5% or 1.5% of agar. Kanamycin at 100 μg/mL and galactose at 2.5% (wt/vol) were added to media for selection when specified. E. coli strains were grown at 37°C on LB agar plates or in LB liquid medium on a rotary shaker at 160 rpm. When necessary, kanamycin was added at 30 μg/mL, chloramphenicol at 20 μg/mL, and ampicillin at 50 μg/mL.

10.1128/mbio.03001-22.10 TABLE S4 Strains, plasmids, and primers used in this study. Download Table S4, DOC file, 0.1 MB. Copyright © 2023 Sourice et al. 2023 Sourice et al. https://creativecommons.org/licenses/by/4.0/ This content is distributed under the terms of the Creative Commons Attribution 4.0 International license.

### Determination of operonic structure.

Total RNA was isolated using the Maxwell 16 miRNA tissue kit from Promega then treated with Turbo DNase TM from Ambion. RNA quality was checked with the TapeStation from Agilent, and the quantification was performed spectrophotometrically. One microgram of total RNA was reverse-transcribed with the GoScriptTM Reverse Transcription System using random hexamers (Promega). cDNA was used as a template in PCRs using the primers described in Table S4 to obtain operon organization.

### Phylogenomic analysis.

The proteome database was previously described and was built to represent the diversity of prokaryotes (9). Briefly, we assembled a proteome database of 553 prokaryotes that include 150 Archaea and 403 Bacteria from NCBI, selecting 1 to 10 representative proteomes per phylum for Bacteria and per major clade in Archaea, depending on the intrataxon diversity (Table S1). The representative genomes have been selected by three criteria: the number of proteins described as reviewed on Uniprot, the completeness status, and the presence of an annotation on the NCBI. The genome of M. xanthus has been added to this selection, leading to the building of a database of 553 proteomes in total.

We then searched proteins of the Rrf2 family by using BLAST v2.8.1+ (66), and E. coli IscR sequence as a seed (AIZ52349.1), with a threshold for the E value at 1e-4. Retrieved sequences have been aligned using MAFFT v7.419 (67) (L-insi option), and the alignment has been manually curated. Then, we built an HMM profile from the alignment using hmmbuild from the HMMER v3.2.1 package (68). The profile has been used to interrogate the database using hmmsearch. We selected the sequences with an E value lower than 0.01. All sequences have been pooled and realigned, and the alignment has been curated (supplemental data available at https://figshare.com/articles/dataset/Sourice_et_al_Supplementary_data/21583104). Then, the alignment has been trimmed using BMGE v1.12 (substitution matrix: BLOSUM30; entropy threshold = 0.95; windows size = 1) (69) selecting the informative positions within the alignment (641 sequences, 135 amino-acid positions; supplemental data). The ML phylogeny has been inferred using IQ-TREE v1.6.10 (70), by selecting the best-suited model according to the BIC criterium (LG+R9) (supplemental data). The robustness of branches has been assessed by 1,000 ultrafast bootstrap replicates. The data set of Fe-S cluster machinery assembly proteins originates from a previous study (9), completed with the homologues detected in the M. xanthus genome. The genomic context has been mapped using GeneSpy and iTOL (71, 72). The supplemental data are available at https://figshare.com/articles/dataset/Sourice_et_al_Supplementary_data/21583104.

### Strains and plasmids construction.

Plasmids used in this study are listed in Table S4. To construct M. xanthus in-frame deletion strains, 800 bp upstream and downstream of the gene(s) targeted for deletion were amplified by PCR and ligated into the pBJ114 following the Hot Fusion protocol (73). Primers used are listed in Table S4. The resulting plasmids (pBJ114-Δ*risR*, pBJ114-Δ*iscU*, pBJ114-Δ*sufBCD*, and pBJ114-Δ*risRsufBCD*) were then introduced into the M. xanthus DZ2 strain by electroporation, and the first homologous recombination was selected on agar plates containing kanamycin. The second homologous recombination allowing gene excision and loss of *galK* was selected on agar plates containing galactose (74).

To construct the Δ*risR risRHis_WT* strain, a PCR fragment containing 1,000 bp upstream of the start codon of *risR* and the *risR* gene was amplified by PCR and ligated into pSWU19 using a T4 DNA ligase. The His tag was encoded by the downstream primer. The resulting plasmid (pSWU19-*P_suf_::risR_*WT) was integrated into the chromosome at the Mx8 phage *attB* site of the Δ*risR* mutant by site-specific integration. The resulting Δ*risR risRHis_WT* strain was selected on kanamycin agar plates. The same protocol was followed to obtain the pSWU19-*P_risR_-risR_3CA* plasmid and the Δ*risR risR_3CA* strain.

To construct the transcriptional *P_suf_::_3_mCherry* fusion, an initial plasmid, pBJ114-*_2_mCherry*, carrying two tandem copies of the *mCherry* gene was constructed. Briefly, two fragments, corresponding to the *mCherry* gene with ribosome binding sites (RBS), were amplified by PCR using the corresponding primers and ligated into a pBJ114 following the Hot Fusion protocol. In parallel, we constructed the transcriptional *P_suf_::mCherry* fusion as follows. PCR fragments corresponding to *P_suf_* (1,000 bp upstream of the start codon of *risR*) and to the promoterless *mCherry* gene without RBS were obtained using the 114PromRisRBamH1_for/PromRisR-mCherry_rev and mCherry-PromRisR_for/114mCherryEcoRI_rev primers pairs, respectively. Both fragments were ligated into the pBJ114 using the appropriate restriction enzyme, leading to the pBJ114-*P_suf_::mCherry* (73). Finally, the pBJ114-*P_suf_::_3_mCherry* was obtained after digestion of the pBJ114-*_2_mCherry* by XbaI and SalI and ligation with the XbaI-SalI fragment carrying the *P_suf_::mCherry* fusion from the pBJ114-*P_suf_::mCherry* plasmid. The pBJ114-*P_suf_::4mut-_3_mCherry* was obtained in a similar manner, except that the XbaI-SalI fragment carrying the *P_suf_::4mut-mCherry* fusion was originated from the pBJ114*-P_suf_::4mut-mCherry* plasmid, which was obtained by site-directed mutagenesis. Plasmid integration in the chromosome of M. xanthus WT and Δ*risR* was selected on kanamycin agar plates. Location of the inserted pBJ114-derived plasmids at the *suf* promoter region was verified by PCR. The same strategy was used to construct M. xanthus strains carrying the *Pisc::3mCherry Pisc::4mut3mCherry* reporter fusions.

Plasmid pET-22b(+) carrying *risR* (pPK14612) was constructed by amplifying by PCR using the pSWU19-*P_risR__risR_WT_* as the template, and the resulting PCR products were digested with EcoRI and XbaI and ligated into the corresponding plasmids.

The expression vector pET11a-RisR was constructed using Gibson assembly. A pET11a vector with an N-terminal Strep-II tag and an enterokinase digestion site (pPK14636) was amplified using primers pET11aF2 and pET11a_NtermStrep. Primers Myxo11 and Myxo08 were used to amplify the *risR* gene from pET-22b(+) carrying the *risR* gene (pPK14612). The resulting fragments were then assembled using the 2X HiFi kit (NEB). The resulting plasmid (pPK14637) was confirmed by DNA sequencing.

For isolation of RisR, a Δ*iscR* derivative of E. coli BL21 was constructed that contained the repaired *suf* operon under the control of the arabinose inducible *P_BAD_* promoter (PK14623) (75). Primers with homology to the *suf* operon were used to amplify a fragment containing *cat* flanked by FRT sites, *araC* and *P_BAD_* promoter region from pPK9125 (19); the fragment was recombined into PK9012 (76 (Beauchene et al, 2017)) by selection with chloramphenicol and then transduced into PK7878 (42) using P1*vir.* The chloramphenicol and kanamycin resistance cassettes were removed using pCP20 (77). Finally, the Rosetta 2 pLysS plasmid (MD Millipore) was transformed into the strain to generate PK14623.

For footprinting assays, plasmid derivatives of pPK7179 (78) containing 300 bp upstream of the start codon of the *isc* or the *suf* operons were constructed using Gibson assembly. The vector was amplified with primers ZM27 and ZM28, and primers Myxo12, Myxo13, Myxo14, and Myxo15 were used to amplify the M. xanthus *isc* and *suf* promoter regions, resulting in pPK14640 and pPK14641, respectively.

### RisR isolation.

Strain PK14639 (PK14623 carrying pPK14637 plasmid) was grown in 1 L of Terrific broth (RPI) with 50 μg/mL ampicillin and 10 mM arabinose, inoculated to 0.5% from an overnight culture to an OD_600nm_ of 1.0, and induced with 0.4 μM IPTG along with the addition of 2 mM cysteine and 0.2 mg/mL of Ferric ammonium citrate. After 1 h, the culture was transferred to 4°C and sparged with argon overnight. All subsequent steps were performed under anaerobic conditions in an anaerobic chamber (Coy Laboratory Products). The cells were harvested, resuspended in lysis buffer (50 mM potassium phosphate buffer, 100 mM NaCl, 10% glycerol, 1 mM DTT, pH 7.2), and lysed using a French press at 20,000 lb/in^2^. The lysate was centrifuged for 1 h at 4°C at 45,000 rpm in a Beckman 70.1 Ti rotor. The supernatent was then loaded onto a 5-mL StrepTrap HP (Cytiva) column equilibrated in the same lysis buffer and connected to a FPLC AKTA Pure system. The protein was eluted using a step gradient with lysis buffer containing 2.5 mM desthiobiotin (EMD Millipore). For measurement of the visible spectrum, the N terminus tagged protein was transferred under anaerobic conditions into a screw top quartz cuvette (Starna). The spectrum was recorded in a Perkin-Elmer λ25 spectrophotometer.

### RNA preparation and reverse transcription.

Ten mL of M. xanthus was grown overnight in casitone-yeast extract (CYE) medium. Cultures were standardized to an OD_600nm_ of 0.2 in CYE medium and supplemented with 20 μM PMS or 150 μM DIP when indicated. After 6 h of growth at 32°C, 1 mL of cells, or 2 mL of cells for the DIP condition, was collected by centrifugation, and cell pellets were frozen at Stick −80°C in liquid N_2_. To isolate RNA, pellets were treated for 3 min with 5 μL of lysozyme (10 mg/mL), and RNA was extracted using the Maxwell 16 LEV miRNA Tissue kit (Promega) according to the manufacturer’s instructions with the inclusion of an extra TURBO DNase (Invitrogen) digestion step to eliminate contaminating DNA. RNA quality was assessed with a TapeStation system (Agilent). RNA was quantified spectrophotometrically at 260 nm (NanoDrop 1000; Thermo Fisher Scientific). For cDNA synthesis, 1 μg total RNA and 0.5 μg random primers (Promega) were used with the GoScript Reverse transcriptase (Promega) according to the manufacturer’s instructions.

### Quantitative real-time PCR for transcriptional analyses.

Quantitative real-time PCR (qPCR) analyses were performed on a CFX96 Real-Time System (Bio-Rad). The reaction volume was 15 μL, and the final concentration of each primer was 0.5 μM. The cycling parameters of the qPCR were 98°C for 2 min, followed by 45 cycles of 98°C for 5 s, 58°C for 10 s, and 72°C for 1 s. A final melting curve from 65°C to 95°C was added to determine the specificity of the amplification. To determine the amplification kinetics of each product, the fluorescence derived from the incorporation of EvaGreen into the double-stranded PCR products was measured at the end of each cycle using the SsoFast EvaGreen Supermix 2X kit (Bio-Rad, France). The results were analyzed using Bio-Rad CFX Maestro software, version 1.1 (Bio-Rad, France). The 16S RNA was used as a reference for normalization. For each sample, a technical duplicate was performed. The amplification efficiencies for each primer pair were between 80 and 100%. Primer pairs are reported in Table S4.

### Site-directed mutagenesis.

Substitution of the Cys92, Cys102, and Cys108 residues from RisR to alanine residues were generated in two steps using first the pSWU19-*P_suf_::risR_WT_* as PCR template and two sets of primer pairs 23/RisRC92A_rev and RisRC92A_for/22 (Table S4). Primers RisRC92A_rev and RisRC92A_for were mutagenic primers and showed overlapping extensions. The two PCR fragments obtained were used for a second PCR run with primers 22 and 23 (Table S4) to obtain complete amplification of the full fragment, which was introduced in the plasmid pSWU19 by T4 DNase ligation. Mutations were confirmed by DNA sequencing. The second step used the newly obtained plasmid as PCR template and the two sets of primers 23/RisRC102AC108A_rev and RisRC102AC108A_for/22 and followed the same protocol as the first step. The same protocol was followed to mutate four nucleotides in the promoter region of the *suf* and *isc* operons by using pBJ114 carrying *P_suf_::mCherry* and *P_isc_::mCherry* as the template.

### Fluorescence quantification with microplate reader.

Overnight cultures of Myxococcus xanthus carrying the fusion 3mCherry and the WT strain were diluted to an OD_600nm_ of 0.2 in 10 mL of CYE in a 100-mL flask. After 6 h of growth at 32°C, 150 μL of culture was dispensed to a microplate Greiner Bio-One 96 well black with transparent bottom and placed in a microplate reader TECAN infinite M200 at 32°C with shaking. The absorbance at 600 nm and the fluorescence of the mCherry with a λ_ex_ of 580 nm and a λ_em_ of 620 nm were measured. After subtracting background absorbance and fluorescence of the CYE media, the fluorescence of mCherry was divided by the OD_600nm_, and this ratio of the WT strain without any mCherry fusion was subtracted from strains carrying a fusion. Finally, these data were normalized to the strain carrying the fusion with the WT promoter of the gene of interest. To avoid a high variability in the results due to the aggregation ability of M. xanthus, values between 0.5 h and 6 h were averaged.

### DNase I footprinting.

Plasmids pPK14640 and pPK14641 were isolated using a QIAfilter maxi kit (Qiagen), and 30 ng was digested using BamHI and HindIII enzymes (NEB) to expose the 3′ ends of the fragment for radiolabeling with [α-^32^P]dGTP (PerkinElmer) by Sequenase (Thermo Fisher). The relevant fragments were separated and isolated from a 5% acrylamide-TBE (Tris-borate-EDTA) gel using a QIAquick gel extraction kit (Qiagen). Promoter fragments were incubated with RisR (1 μM) for 25 min in 25 mM potassium phosphate buffer, 30 mM KCl, 5 mM potassium glutamate, 100 μg/mL BSA, and 1 mM DTT at 37°C under anaerobic conditions. DNase I (Worthington) 2 μg/mL in 65 mM MgCl_2_ was added for 30 s, and the reaction was terminated with 300 mM acetate and 20 mM EDTA, ethanol precipitated and resuspended in 4 μL urea loading dye, heated to 90°C for 1 min before loading a 7-M urea −8.0% polyacrylamide gel in 0.5× TBE buffer. The G+A ladder for each radiolabeled promoter fragment was achieved by DNA modification using formic acid followed by piperidine cleavage (79). The gel was visualized in an Amersham Typhoon 5-gel imaging scanner (Cytiva). For some experiments, 5 μM RisR was treated with 32 units of enterokinase (NEB) to remove the N-terminal tag by 2 h of incubation at room temperature under anaerobic conditions in 50 mM potassium phosphate buffer, 100 mM NaCl, 2 mM CaCl_2_, and 10% glycerol pH 7.2.

### Growth curves of M. xanthus and mobility assay on agar plates.

Overnight cultures of M. xanthus at 32°C in CYE medium were diluted to an OD_600nm_ of 0.1 in 20 mL of CYE supplemented when specified with 10 μM PMS or 150 μM DIP. Then, the cultures were grown at 32°C under agitation at 160 rpm. Growth curves were obtained by measuring absorbance at 600 nm. Motility was tested using overnight M. xanthus cultures grown at 32°C in CYE medium and concentrated to an OD_600nm_ of 5 in CYE. Ten microliters of cells were spotted onto CYE 0.5% agar plates supplemented or not with 10 μM PMS or 100 μM DIP. Agar plates were incubated 48 h at 32°C. Pictures of the colonies were taken using the Stereo Microscope Nikon SMZ745T binocular loupe equipped with a Nikon Camera.

### Membrane fractionation.

M. xanthus strains were grown on CYE medium (500 mL) to an OD_600nm_ between 0.4 and 0.6. When specified, 10 μM PMS or 150 μM DIP were added at this OD_600nm_ and the cultures were grown at 32°C for 6 h. The cells were then harvested, and the pellets were resuspended in 20 mL of buffer M composed of 10 mM Tris-HCl pH 7.6, 1 mM KH_2_PO_4_, and 8 mM MgSO_4_. In addition, 0.1 mg/mL of DNase I and 10 mM MgCl_2_ were added to facilitate cell lysis. The cells were mechanically lysed by a high-pressure homogenization system using an Emulsiflex C5 AVESTIN. The lysate was centrifuged to eliminate remaining cells, and the supernatant was centrifuged 1 h at 40,000 rpm in a Type 70 Ti Fixed-Angle Titanium to pellet the membranes, which were resuspended in 200 μL of the buffer M.

### O_2_ consumption measurements.

O_2_ consumption measurements were performed by polarography with a Clark type electrode at 32°C. A membrane suspension was added to the oxygraph chamber, which contained 1.8 mL of air-saturated buffer M, and the O_2_ concentration variation was recorded as a function of time. One hundred percent air saturation corresponded to 228 μM O_2_ dissolved at 32°C. The rate of O_2_ consumption of the membranes was measured by adding 16 mM succinate.

### Protein concentration determination.

Quantification of N terminus tagged RisR and total proteins in the membranes was carried out by the Bradford assay using BSA as the standard.

### Western blot analysis.

Overnight cultures grown in CYE medium were used to inoculate fresh CYE medium to an OD_600nm_ of 0.2. Strains were grown 6 h at 32°C under shaking, and 1 mL of OD_600nm_ 0.5 of cells was pelleted. Then, 50 μL of Laemmli buffer and 50 μL of water were added and heated for 9 min at 95°C. Ten microliters were electrophoresed onto a 15% acrylamide/bis-acrylamide gel and then transferred to a nitrocellulose blotting membrane, Amersham Protan 0.45 μm NC. To assay the production of 6His tagged versions of RisR, the membrane was washed with PBS-Tween 0.05%, followed by 1 h of blocking in PBS-BSA 3% at room temperature and washed again before adding the Penta-His Antibody (Qiagen) diluted at 1:1,000 in PBS-BSA 3%, which was used as the primary antibody at 4°C overnight. Then, an anti-mouse IgG (HRP) (Promega) was used as a second antibody diluted at 1:10,000 in PBS-Tween 0.05% and applied 1 h at room temperature. Signal was detected with the SuperSignal West HisProbe kit (Thermo Scientific) and an ImageQuant LAS 4000 mini.
