# Supplementary figures and images for: A Diverged Transcriptional Network for Usage of Two Fe-S Cluster Biogenesis Machineries in the Delta-Proteobacterium Myxococcus xanthus
Source: mBio. 2023 Jan 19;14(1):e03001-22. doi: 10.1128/mbio.03001-22 (PMC9973013; doi:10.1128/mbio.03001-22)

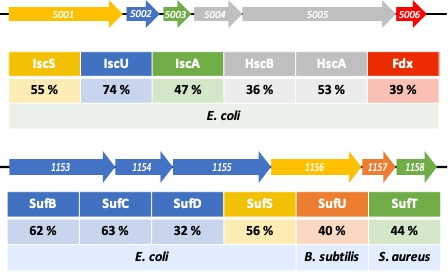

Supplement: FIG S1 [file mbio.03001-22-s0001.tif]

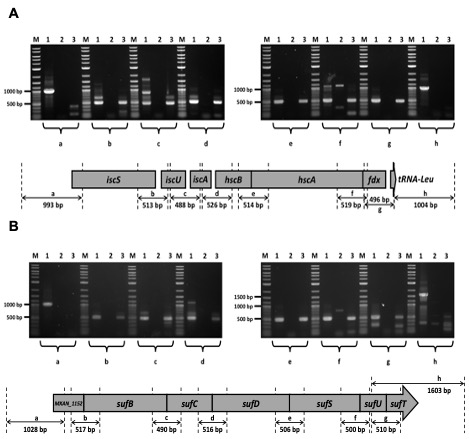

Supplement: FIG S2 [file mbio.03001-22-s0002.tif]

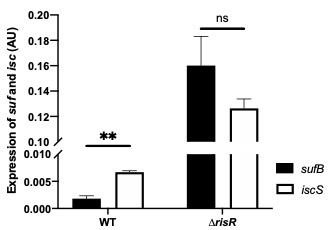

Supplement: FIG S3 [file mbio.03001-22-s0006.tif]

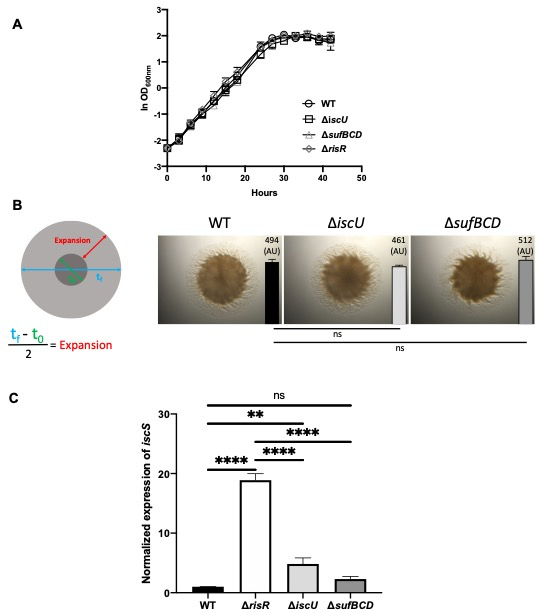

Supplement: FIG S4 [file mbio.03001-22-s0007.tif]

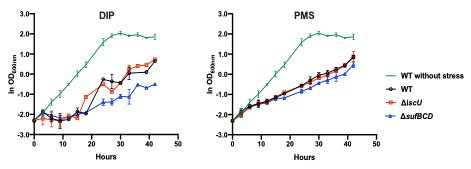

Supplement: FIG S5 [file mbio.03001-22-s0008.tif]

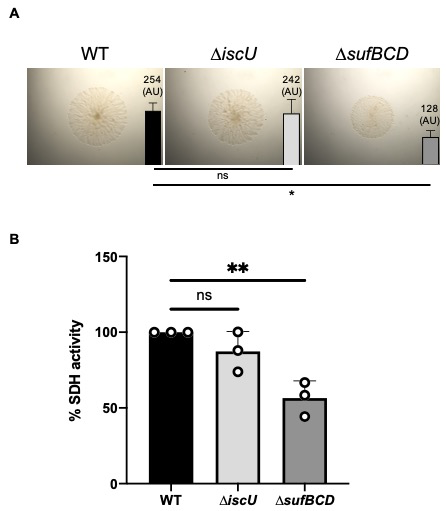

Supplement: FIG S6 [file mbio.03001-22-s0009.tif]
