# Supplementary material for: A Diverged Transcriptional Network for Usage of Two Fe-S Cluster Biogenesis Machineries in the Delta-Proteobacterium Myxococcus xanthus
Source: mBio. 2023 Jan 19;14(1):e03001-22. doi: 10.1128/mbio.03001-22 (PMC9973013; doi:10.1128/mbio.03001-22)
Supplement: TABLE S4 [file mbio.03001-22-s0010.docx]

| Table S4. Strains, plasmids and primers used in this study   \| Strain \| Relevant Genotype (comments) \| Reference \| \| --- \| --- \| --- \| \| *M. xanthus* \|  \|  \| \| DZ2 \| Wild type strain \| (1) \| \| *∆risR* \| DZ2 ∆*risR* \| This study \| \| ∆*iscU* \| DZ2 ∆*iscU* \| This study \| \| ∆*sufBCD* \| DZ2 ∆*sufBCD* \| This study \| \| ∆*risR* ∆*iscU* \| DZ2 ∆*risR* ∆*iscU* \| This study \| \| ∆*risR* ∆*sufBCD* \| DZ2 ∆*risR* ∆*sufBCD* \| This study \| \| *P_suf_::_3_mCherry* \| DZ2 *pBJ114-P_suf_::_3_mCherry* \| This study \| \| *P_isc_::_3_mCherry* \| DZ2 *pBJ114-P_isc_::_3_mCherry* \| This study \| \| *P_suf_::4mut-_3_mCherry* \| DZ2 *pBJ114-P_suf_::4mut-_3_mCherry* \| This study \| \| *P_isc_::4mut-_3_mCherry* \| DZ2 *pBJ114-P_isc_::4mut-_3_mCherry* \| This study \| \| *∆risR P_suf_::_3_mCherry* \| DZ2 *∆risR pBJ114-P_suf_::_3_mCherry* \| This study \| \| *∆risR P_isc_::_3_mCherry* \| DZ2 *∆risR pBJ114-P_isc_::_3_mCherry* \| This study \| \| ∆*risR risRHis_WT* \| DZ2 ∆*risR att_Mx8_::pSWU19-P_suf_::risR* \| This study \| \| ∆*risR risR_3CA* \| DZ2 ∆*risR att_Mx8_::pSWU19-P_suf_::risR^C92A/C102A/C108A^* \| This study \| \| *E. coli* \|  \|  \| \| PK9012 \| MG1655 Δ*mutS::Tn10 λc1857* Δ*cro-bioA* \| (2) \| \| PK7878 \| BL21(DE3) ΔhimA∷tet ΔiscR∷kan \| (3) \| \| PK14623 \| BL21(DE3) ΔhimA∷tet ΔiscR::FRT FRT-araC- P_BAD ::_suf operon + Rosetta^TM^ 2 plysS \| This study \| \| PK14639 \| PK14623 carrying the pPK14637 plasmid \| This study \| | | |
| --- | --- | --- | --- | --- | --- | --- | --- | --- | --- | --- | --- | --- | --- | --- | --- | --- | --- | --- | --- | --- | --- | --- | --- | --- | --- | --- | --- | --- | --- | --- | --- | --- | --- | --- | --- | --- | --- | --- | --- | --- | --- | --- | --- | --- | --- | --- | --- | --- | --- | --- | --- | --- | --- | --- | --- | --- | --- | --- | --- | --- | --- | --- | --- | --- | --- |
| Plasmid | Relevant Genotype | Reference |
| pBJ114 | Used to create deletions or insertions, *galK,* KanR | Laboratory collection |
| pBJ114-∆*risR* | pBJ114 with deletion cassette for *risR* | This study |
| pBJ114-∆*iscU* | pBJ114 with deletion cassette for *iscU* | This study |
| pBJ114-∆*sufBCD* | pBJ114 with deletion cassette for *sufBCD* | This study |
| pBJ114-∆*risRsufBCD* | pBJ114 with deletion cassette for *risR* and *sufBCD* | This study |
| pBJ114-*P_suf_::_3_mCherry* | pBJ114 with insertion cassette for the creation of *P_suf_::3mCherry* | This study |
| pBJ114-*P_suf_::4mut-_3_mCherry* | pBJ114 with insertion cassette for the creation of *P_suf_::*(G/T, G/T, A/C, C/T)-*_3_mCherry* | This study |
| pBJ114-*P_isc_::_3_mCherry* | pBJ114 with insertion cassette for the creation of *P_isc_::_3_mCherry* | This study |
| pBJ114-*P_isc_::4mut-_3_mCherry* | pBJ114 with insertion cassette for the creation of *P_isc_::* (G/T, A/C, C/A, C/A)*-_3_mCherry* | This study |
| pSWU19 | Used for insertions at Mx8 phage *attB* site, KanR | Laboratory collection |
| pSWU19-*P_suf_::risRHis_WT* | pSWU19 to express WT RisR his-tagged from its native promoter (*P_suf_::iscR_WT)* at the Mx8 phage *attB* site | This study |
| pSWU19*-P_suf_::risRHis^C92A/C102A/C108A^* | pSWU19 to express RisR3CA his-tagged from its native promoter (*P_suf_::risR^C92A/C102A/C108A^*) at the Mx8 phage *attB* site | This study |
| pPK14612 | pET-22b(+) with *risR* | This study |
| pPK14636 | pET11a StrepII Tag- enterokinase site | Kiley lab |
| pPK14637 | pET11a StrepII Tag- enterokinase site in frame with *risR* | This study |
| pPK9125 | FRT–*cat*–FRT from pKD32 cloned into *Sph*I site of pBAD/Myc–hisC | (4) |
| pCP20 | yeast Flp recombinase gene | (5) |
| pPK7179 | pUC19-*spf* with *Xho*I site replacing *Sal*I site | (6) |
| pPK14640 | pPK7179 with *Pisc* | This study |
| pPK14641 | pPK7179 with *Psuf* | This study |

| Primer | Sequence (5’-3’) | Used for |
| --- | --- | --- |
| promRisR_HindIII_for_bis | ACAGCTATGACATGATTACAAGCTTCCATGGAGAACGCGCTCTTGTTGTCG | pBJ114-∆*risR* |
| RisR-SufB-rev | GTTCCTGGAGGGTTTCGGTGCTCATGGGTGCGGGTGTCTCCTCTCTGGG |  |
| RisR-SufB_for | CCCAGAGAGGAGACACCCGCACCCATGAGCACCGAAACCCTCCAGGAAC |  |
| SufB_XbaI_rev | GCTCGGTACCCGGGGATCCTCTAGACTGCACCGTGGAGTACTTGATGGTG |  |
| IscS_HindIII_for | ACAGCTATGACATGATTACAAGCTTCCAAGGGCGTGCTCTTCCAC | pBJ114-∆*iscU* |
| IscS_IscA_rev | GGTTCGCCTCTCCCTTTCCCGAACGGCCTGCTCCTTCAACGGATGCTTCT |  |
| IscS_IscA_for | AGAAGCATCCGTTGAAGGAGCAGGCCGTTCGGGAAAGGGAGAGGCGAACC |  |
| IscA_XbaI_rev | GCTCGGTACCCGGGGATCCTCTAGACCTTCTGCGCGCCCGCGTCC |  |
| RisR_HindIII_for | ACAGCTATGACATGATTACAAGCTTGTCGCCTGCCCCTCCGGCGAGGGCG | pBJ114-∆*sufBCD* |
| RisR_SufU_rev | CGGACCTGATTCACGTCGAAGCCGCTCATGAGCGGACTCCTGTCGCGGAA |  |
| RisR_SufU_for | TTCCGCGACAGGAGTCCGCTCATGAGCGGCTTCGACGTGAATCAGGTCCG |  |
| SufU_XbaI_rev | GCTCGGTACCCGGGGATCCTCTAGAGTTGTACGTCACCTTCTCCA |  |
| PrisR-SufS_rev | CCTGATTCACGTCGAAGCCGCTCATGGGTGCGGGTGTCTCCTCTCTGGGT | pBJ114-∆*risRsufBCD* |
| PrisR-SufS_for | ACCCAGAGAGGAGACACCCGCACCCATGAGCGGCTTCGACGTGAATCAGG |  |
| 114PromRisRBamH1_for | CAGGTCGACTCTAGAGGATCCCCAGCCGTCGCCGCCGAGCAG | pBJ114-*P_suf_::mCherry* |
| PromRisR-mCherry_rev | GTTATCCTCCTCGCCCTTGCTCACCATGGGTGCGGGTGTCTCCTCTCTGGGTC |  |
| mCherry-PromRisR_for | GACCCAGAGAGGAGACACCCGCACCCATGGTGAGCAAGGGCGAGGAGGATAAC |  |
| 114McherryEcoRI_rev | GTAAAACGACGGCCAGTGCCGAATTCTTACTTGTACAGCTCGTCCATG |  |
| promIscBamHI_for Bis | CCTGCAGGTCGACTCTAGAGGATCCCGCACGTCCTGGATTTCAATCG | pBJ114-*P_isc_::mCherry* |
| PromIscSMcherry_rev | GTTATCCTCCTCGCCCTTGCTCACCATTGCTTCCAACACCTTCCTGGAGGGG |  |
| mCherrypromIscS_for | CCCCTCCAGGAAGGTGTTGGAAGCAATGGTGAGCAAGGGCGAGGAGGATAAC |  |
| 22 | AGCGGATAACAATTTCACACAGGA | Site directed mutagenesis of *P_suf_* and *P_isc_* |
| 23 | CGCCAGGGTTTTCCCAGTCACGAC |  |
| PrisR_4mut_rev | TTCAAGAAACATTGCAATTTGAATCCCTCC |  |
| PrisR_4mut_for | ATGTTTCTTGAAACGGTCCTGATTACATAT |  |
| PiscS_4mut_rev | ACTTTGAAAATATAAAAAGGGGCGCCTTCG |  |
| PiscS_4mut_for | TATTTTCAAAGTCCGGTCCACATTCAGGTG |  |
| mCherry2_SalI_XbaI_114_for | AGCTCGGTACCCGGGGATCCTCTAGAAAGAAAGAGTCGACAGGATGAGGATCGTTTCGC | pBJ114-*_2_mCherry* |
| mCherry2_mCherry3_rev | TTCTTACACTCCGGATTCGCGAAAATTTACTTGTACAGCTCGTCCATGCCGC |  |
| mCherry2_mCherry3_for | ATTTTCGCGAATCCGGAGTGTAAGAAATGGTGAGCAAGGGCGAGGAGGATAA |  |
| 114_HindIII_mCherry3_rev | AACAGCTATGACATGATTACAAGCTTTTACTTGTACAGCTCGTCCATGCCGC |  |
| 114_XbaI_PromRisR_for | AGCTCGGTACCCGGGGATCCTCTAGACCAGCCGTCGCCGCCGAGCAGCGGCA | pBJ114-*_3_mCherry* |
| PiscS_XbaI_114_for | AGCTCGGTACCCGGGGATCCTCTAGACGCACGTCCTGGATTTCAATCGTGTC |  |
| mCherry1_SalI_mCherry2_rev | TGCGAAACGATCCTCATCCTGTCGACTTACTTGTACAGCTCGTCCATGCCGC |  |
| promRisR_HindIII | TAGCAAGCTTACCTGGCGGTGAACACGTCCGCC |  |
| RisRCterHis_XbaI | TAGCTCTAGATCAGTGGTGGTGGTGGTGGTGTGAGCGGACTCCTGTCGCGG |  |
| RisRC92A_rev | CGCCGGCCTCCGTGAGGGCGACCGGCCC | pSWU19*-P_suf_::risR^C92A/C102A/C108A^* |
| RisRC92A_for | GAGGCCGGCGTCCACACCTCCGGGGGTGCG |  |
| RisRC102AC108A_rev | CTGGGCGACGGACTCCAGCTCGGCGGGCGCACCCCCGGAGGTGTGG | pSWU19*-P _suf_::risR^C92A/C102A/C108A^* |
| RisRC102AC108A_for | CCCGCCGAGCTGGAGTCCGTCGCCCAGGTGCGGGGCCACTGGCGCCTCATC |  |
| NterIscR_EcoRI | GATCGAATTCATGCTCCGGATGAGCAAGATG | pPK14612 |
| CterIscR_XbaI_rev | TCGACTCTAGATCATGAGCGGACTCCTGT |  |
| pET11aF2 | CTAACAAAGCCCGAAAGG | pPK14637 |
| pET11a_NtermStrep | TCTGCACCCTTTTCGAAC |  |
| Myxo11 | CAGTTCGAAAAGGGTGCAGATGACGACGACAAGCTCCGGATGAGCAAGATG | pPK14637  pPK7179 |
| Myxo08 | GGCCCTTTCGTCTTCAAGAAGTCCTCGTCAACCACTCG |  |
| ZM27 | GGATCCCCACAGAGGTAAG |  |
| ZM28 | CTCGAGCTGCAGGCATGC |  |
| Myxo12 | TTGCATGCCTGCAGCTCGAGGCCAGCGAGGGGCCTGCC | pPK14640 |
| Myxo13 | TCTTACCTCTGTGGGGATCCTGCTTCCAACACCTTCCTGGAGGGG |  |
| Myxo14 | TTGCATGCCTGCAGCTCGAGGCGGCCGGTACCCCGGTC | pPK14641 |
| Myxo15 | TCTTACCTCTGTGGGGATCCGGGTGCGGGTGTCTCCTCTC |  |

**References**

1. Campos JM, Zusman DR. 1975. Regulation of development in *Myxococcus xanthus*: effect of 3’:5’-cyclic AMP, ADP, and nutrition. Proc Natl Acad Sci U S A 72:518–522.

2. Beauchene NA, Mettert EL, Moore LJ, Keleş S, Willey ER, Kiley PJ. 2017.

O_2_ availability impacts iron homeostasis in *Escherichia coli*. Proc Natl Acad Sci U S A 114:12261–12266.

3. Rajagopalan S, Teter SJ, Zwart PH, Brennan RG, Phillips KJ, Kiley PJ. 2013. Studies of IscR reveal a unique mechanism for metal-dependent regulation of DNA binding specificity. Nat Struct Mol Biol 20:740–747.

4. Giel JL, Nesbit AD, Mettert EL, Fleischhacker AS, Wanta BT, Kiley PJ. 2013. Regulation of iron-sulphur cluster homeostasis through transcriptional control of the Isc pathway by [2Fe-2S]-IscR in *Escherichia coli*. Mol Microbiol 87:478–492.

5. Datsenko KA, Wanner BL. 2000. One-step inactivation of chromosomal genes in Escherichia coli K-12 using PCR products. Proc Natl Acad Sci U S A 97:6640–6645.

6. Kang Y, Weber KD, Qiu Y, Kiley PJ, Blattner FR. 2005. Genome-wide expression analysis indicates that FNR of Escherichia coli K-12 regulates a large number of genes of unknown function. J Bacteriol 187:1135–1160.
